# Pitolisant Inhibits Alcohol Drinking and Improves Withdrawal Negative Affect Through Lateral Habenula Histaminergic Signaling in Mice

**DOI:** 10.1002/cns.70732

**Published:** 2026-01-06

**Authors:** Yan Zhao, Yixin Fu, Tianhao Liu, Zanhao Yang, Zhengzhong Yang, Bingqing Chen, Lipeng Zhou, Juntao Yang, Duo Chen, Xiaojiao Han, Ying Tang, Jiang‐Hong Ye, Chao‐Yu Miao, Rao Fu

**Affiliations:** ^1^ Department of Pharmacology Naval Medical University/Second Military Medical University Shanghai China; ^2^ Department of Anatomy, School of Medicine Shenzhen Campus of Sun Yat‐Sen University, Sun Yat‐Sen University Shenzhen Guangdong China; ^3^ Department of Anesthesiology The Seventh Affiliated Hospital, Sun Yat‐Sen University Shenzhen China; ^4^ Clinical Skills Training Center, School of Medicine Shenzhen Campus of Sun Yat‐Sen University, Sun Yat‐Sen University Shenzhen Guangdong China; ^5^ Department of Anesthesiology, Pharmacology, Physiology & Neuroscience, Rutgers The State University of New Jersey, New Jersey Medical School Newark New Jersey USA; ^6^ Shenzhen Key Laboratory for Systems Medicine in Inflammatory Diseases, School of Medicine Shenzhen Campus of Sun Yat‐Sen University, Sun Yat‐Sen University Shenzhen China

**Keywords:** alcohol use disorder, alcohol withdrawal, histamine H3 receptor (H3R), lateral habenula (LHb), negative mood, pitolisant

## Abstract

**Background:**

Alcohol use disorder (AUD) is a chronic condition marked by compulsive drinking and withdrawal‐related negative affect. Histamine (HA) signaling, particularly via the histamine H3 receptor (H3R), may modulate alcohol‐related behaviors. We investigated the effects of pitolisant, an FDA‐approved H3R antagonist, on ethanol (EtOH)‐related behaviors in mice.

**Method:**

Adult male C57BL/6J mice underwent acute or chronic (2 or > 8 weeks) intermittent alcohol exposure. Pitolisant pretreatment was administered, and then pharmacological behavior, histologic, and molecular assays were conducted.

**Result:**

Pitolisant administration reduced acute EtOH‐induced locomotor activation, conditioned place preference, and sedative effects, and also curtailed EtOH intake. It alleviated anxiety and depression‐like behavior during 24‐h withdrawal (Post‐EtOH). Mechanistically, the Post‐EtOH condition was featured by complicated brain cFos expression mapping, including elevated cFos, [HA] and [glutamine]/[glutamate] ratio in the lateral habenula (LHb). However, systemic pitolisant treatment significantly increased [norepinephrine]/[normetanephrine] ratio, and restored the diminished phosphorylated CREB and BDNF levels in the LHb. Intra‐LHb H2R antagonist cimetidine infusion partly blocked the pitolisant therapeutic effect on alcohol‐related behavior.

**Conclusion:**

These findings highlight the HAergic system as a critical regulator of alcohol‐related behaviors. The LHb HA signaling and norepinephrine neurotransmission might underlie pitolisant's potential novel therapeutic strategy for AUD.

## Graphical abstract

Pitolisant, an FDA‐approved H3R antagonist, counteracts the rewarding and sedative effects of ethanol, reduces alcohol intake, and alleviates the negative affect during withdrawal. This might occur via the antagonism of presynaptic autoreceptors and heteroreceptors H3Rs in the lateral habenula, thereby activating the H2R/p‐CREB/BDNF signaling and modulating norepinephrine neurotransmission.

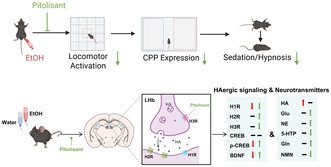

## 1 Introduction

Alcohol use disorder (AUD) is a chronic relapsing condition marked by compulsive alcohol consumption, loss of control, and withdrawal‐induced negative affect [1]. Current FDA‐approved treatments, such as naltrexone and acamprosate, offer limited efficacy and fail to adequately address affective symptoms, highlighting the need for novel therapeutic targets [2].

Histamine (HA), a neurotransmitter involved in sleep–wake regulation, feeding, and cognition [3, 4] is synthesized by histidine decarboxylase (HDC) in the tuberomammillary nucleus (TMN) and widely distributed throughout the brain [5].

Alcohol influences HA levels centrally and peripherally, promoting HA release and inhibiting its degradation, leading to elevated tissue levels [6, 7, 8]. Recent metabolic profiling in AUD patients revealed increased HA turnover, suggesting altered HAergic signaling.

Preclinical studies have shown that HA modulates alcohol reward. HDC‐knockout mice exhibit increased alcohol preference [6], and alcohol‐preferring rats have higher brain HA levels [9]. Notably, antagonism or genetic deletion of H3R, a presynaptic autoreceptor that regulates HA and other neurotransmitter release, reduces alcohol consumption and seeking behavior in rodents [10, 11, 12, 13].

Pitolisant, the first FDA‐approved H3R inverse agonist for narcolepsy [14, 15], enhances HAergic tone and raises dopamine (DA) and acetylcholine (ACh) levels in key brain regions [16]. Its wide‐ranging neuropharmacological profile implies potential uses beyond sleep disorders, such as in epilepsy [17], Parkinson's disease [18], and neuropsychiatric conditions [19, 20, 21, 22]. It has distinct advantages over other H3R antagonists, making it a promising candidate for alcohol‐related behavior research. With a non‐imidazole structure, it is highly selective for H3R, has minimal CYP450 interaction, and thus has a low drug–drug interaction risk [23]. Clinically, it has good pharmacokinetics, low abuse potential, and a well‐characterized safety profile, which is beneficial for AUD repurposing [24]. However, its role in alcohol‐related behaviors and mechanism remains to be explored.

The lateral habenula (LHb), a brain region implicated in aversion and mood regulation, receives dense HAergic input and expresses high levels of H3R [25, 26]. Hyperactivity in the LHb and its downstream targets, such as the rostromedial tegmental nucleus (RMTg), contributes to alcohol dependence and withdrawal‐induced negative affect [27, 28, 29, 30, 31]. Based on this, we hypothesize that pitolisant may reduce alcohol consumption and alleviate withdrawal negative mood state via H3R blockade in the LHb.

This study explored the therapeutic potential of pitolisant for alcohol‐related behaviors in mice. We evaluated its effects on ethanol (EtOH) induced reward, consumption, and withdrawal negative affect, and investigated the underlying neural mechanisms in the LHb. Here, we report that pitolisant significantly counteracted the rewarding property of alcohol, reduced alcohol consumption, and demonstrated an anxiolytic and anti‐depressant effect in withdrawn mice. The histamine H2 receptor signaling in the LHb and norepinephrine neurotransmission might be involved in pitolisant's action.

## 2 Materials and Methods

### 2.1 Animals

The C57BL/6J mice (aged 8–10 weeks at the start of the experiments) were obtained from the Laboratory Animal Center of Sun Yat‐sen University and used in the investigation. See Table S1 for a full subject description. All procedures were performed according to the Chinese National Health and Medical Research Council animal ethics guidelines and with the approval of the Sun Yat‐sen University Animal Experimentation Ethics Committee. Mice were housed individually in home cages in a climate‐controlled room (20°C) under a 12‐h light/dark cycle. Animals were allowed to acclimate to the housing conditions and handling before the start of each experiment. Food and water were available *ad libitum*.

### 2.2 Drugs

The following pharmacological antagonists were employed: pitolisant (HY‐12199B, MCE, USA), cimetidine (Sigma‐Aldrich, C4522, USA), and triprolidine (Sigma‐Aldrich, T6764, USA). Cimetidine was dissolved in 0.15 M hydrochloric acid with artificial cerebrospinal fluid (aCSF), and the solution was then adjusted to a neutral pH using 1 M sodium hydroxide. All other drugs were dissolved in either 0.9% saline solution or aCSF. The drugs were administered either through unilateral intracerebroventricular (i.c.v) injection (200 nL) or bilateral microinfusion into the lateral habenula (LHb, 200 nL). The systemic injection doses of pitolisant (5 and 10 mg/kg) and its microinfusion dose (5 μg/200 nL/side) were selected based on a previous study. A 10 mg/kg dose was found to effectively elevate the brain histamine level without causing adverse effects [32]. The triprolidine and cimetidine doses were chosen considering their capacity to block histamine responses [33]. All other chemical information was listed in the Supplementary File.

### 2.3 Two‐Bottle Choice Drinking Procedure

The effects of either acute or chronic pitolisant administration on voluntary EtOH consumption were assessed in C57BL/6J mice subjected to a two‐bottle choice paradigm. Mice were habituated to drink from either a water bottle or a bottle containing a 3% (days 1–4) or a 6% (days 5–8) EtOH solution (*v/v*), which was placed in their home cage. On day 8, mice received a single vehicle or pitolisant administration at 9:00 a.m., and the bottles were weighed at intervals of 0, 90 min, 4 h, and 24 h to assess fluid consumption.

### 2.4 Intermittent Access to 20% EtOH in the Two‐Bottle Free Choice (IA2BC) Drinking Procedure

We assessed the effects of acute and chronic pitolisant administration on voluntary EtOH consumption in mice using an IA2BC procedure, as described in previous studies [34]. The procedure entailed giving the mice 24‐h concurrent access to two bottles, one filled with 20% EtOH (v/v) and the other with water, commencing at 9:00 a.m. on Mondays. After 24 h, the EtOH bottle was swapped with a second water bottle. This pattern was replicated on Wednesdays and Fridays. On all other days, the mice had unrestricted access to two water bottles. To control for any side preferences, the position of the EtOH bottle was alternated between each drinking session.

The quantity of EtOH and water consumed was gauged by weighing the bottles before and after each 24‐h access period. EtOH intake was computed in grams of alcohol per kilogram of body weight. Weekly “drip” averages (fluid lost from the cages without animals' presence) were deducted from the individual fluid intake. Spillage was consistently less than 1.0 mL (or less than 2.5% of total fluid intake) over the 24‐h period. All mice were weighed weekly.

### 2.5 Behavior Assays

The loss of righting reflex (LORR) test was used to evaluate the effect of pitolisant on EtOH‐induced sedation. The open field test (OFT) and conditioned place preference (CPP) test were used to assess how pitolisant influenced EtOH‐induced locomotion activation and reinforcing effects. A battery of behavioral assays was employed to assess the negative affect state associated with EtOH withdrawal. Specifically, the OFT and elevated‐plus maze test (EPM) were used to evaluate anxiety‐like behaviors, while the tail suspension test (TST) and sucrose preference test (SPT) were used to measure depression‐like behaviors in mice 24 h after withdrawal (Post‐EtOH) from IA2BC drinking sessions. From an operational perspective, we defined this time without alcohol consumption as “withdrawal.” Nevertheless, we understand that this term typically describes the symptoms exhibited by alcohol‐dependent patients. Naïve mice served as the control group. The exact conditions of the following assays are described in the Supporting Information.

### 2.6 Stereotaxic Surgery and Microinjection Procedure

Mice were subjected to surgery using a stereotaxic apparatus (68,025, RWD Life Science, Shenzhen, China) while under anesthesia, induced by ketamine/xylazine (80/20 mg/kg, i.p.) and maintained with isoflurane (0.5%–1%) as previously reported [34, 35]. The guide cannula (outer diameter: 0.48 mm, inner diameter: 0.34 mm, 26 G/M3.5, RWD) was unilaterally implanted into the right lateral ventricle (AP: −0.22 mm, ML: 1.1 mm, DV: −2.2 mm from the skull surface) or bilaterally implanted 1 mm dorsal to the LHb (AP: −1.5 mm, ML: ±0.4 mm, DV: −1.7 mm from the skull surface). The cannulas were secured on the skull using jeweler's screws and dental cement. The animals resumed alcohol consumption in the IA2BC procedure 1 week after the surgery.

The mice received chemicals or vehicle control in 200 nL/side through a 26‐gauge internal cannula (1 mm below the cannula placement) connected to the Hamilton 1.0 μL syringe driven by a syringe pump (R462, RWD Life Science, China) at the rate of 100 nL/min, 15 min before the behavioral test [30]. After completing the injection procedure, the injectors were left in place for an additional 60 s to allow for diffusion and prevent reflux of the compound into the cannula.

To verify the injection sites, we selected 4–5 series of sections from each animal, and coronal sections of 30 μm were mounted on slides and stained with Cresyl violet. The injector's placement was determined through a BX‐63 microscope with a 10× objective lens.

### 2.7 Immunofluorescence

We performed cFos and histamine receptors immunofluorescence to evaluate the effect of central administration of pitolisant on neuronal activation of brain regions that are reported to be involved in negative mood and alcohol addiction.

The mice were euthanized with sodium pentobarbital (50 mg/kg, i.p.) and transcardially perfused with ice‐cold saline followed by 4% paraformaldehyde. Brains were removed and post‐fixed in 4% paraformaldehyde for 24 h and transferred to 30% sucrose in phosphate‐buffered solution for 48 h. Serial 30 μm coronal sections of the midbrain were cut on a freezing microtome (NX50, Thermo Fisher Scientific, USA) and protected in a cryoprotectant solution, stored at −20°C until further processing for immunostaining. The immunofluorescence was processed as in previous reports to evaluate the brain cFos and histamine receptors expression in the neurons [30, 36]. See detailed procedure and antibodies information (listed in Table S2) in the Supporting Information.

### 2.8 Western Blot Assays

After the final behavioral test, the mouse was sacrificed under deep anesthesia with isoflurane. Then, transcardial ice‐cold saline perfusion was performed, and the mouse was decapitated for rapid brain dissection. Subsequently, the mouse brain was cut with a vibratome into ice‐cold artificial cerebrospinal fluid (aCSF) containing (in mM): 126 NaCl, 2.5 KCl, 1.25 NaH_2_PO_4_, 1 MgCl_2_, 2 CaCl_2_, 25 NaHCO_3_, 1 L‐ascorbate, and 11 glucose, and saturated with 95% O_2_/5% C_2_ (carbogen).

Tissue containing the LHb was harvested from three to four 400‐μm‐thick coronal slices on ice by punching them out with a stainless‐steel cannula (Brain Punch Set, 57,401, Stoelting Co., IL, USA). Tissue harvested from three mice was pooled and served as each sample in the molecular experiments. See detailed protocols and antibodies information (listed in Table S2) in Supporting Information.

### 2.9 LC–MS Analysis for Neurotransmitters and Metabolites

To assess the impact of Post‐EtOH condition and pitolisant pretreatment on LHb neurotransmitters (NTs) level in mice, we harvested LHb‐containing brain tissue from Post‐EtOH mice to measure 18 NTs and metabolites [glycine (Gly), GABA, HA, tyramine, ACh, glutamine (Gln), glutamate (Glu), dopamine (DA), 3‐methoxytyramine (3‐MT), norepinephrine (NE), serotonin (5‐HT), epinephrine (Epi), metanephrine (MN), 5‐Hydroxyindole‐3‐acetic acid (5‐HIAA), DOPA, 5‐hydroxy‐L‐tryptophan (5‐HTP), thyroxine(T_4_), and normetanephrine (NMN)]. Thirty Post‐ETOH mice were treated with either pitolisant (10 mg/kg, i.p.) or saline, and tissue was collected 30 min post‐administration. LHb tissue from 15 naïve mice served as a control. Tissue from three mice was pooled into one sample. Five samples per group were sent for LC–MS measurement. Sample preparation and LC/MS analysis followed our recent report [7]. See detailed assay conditions in the Supporting Information.

### 2.10 Statistical Analysis

All data are presented as mean ± SEM (standard error of the mean). Animals were randomly assigned to different studies, and investigators were blinded to group allocations in pharmacological behavioral experiments. For evaluating the pharmacological effect of systemic or local administration of pitolisant in a particular brain region on the drinking and affective disorder behavioral tests between naïve and acute EtOH treatment or EtOH withdrawal groups, one‐way or two‐way repeated measures (RM) ANOVA, followed by Bonferroni post hoc test for multiple comparisons, was used. All data passed the check for normality and variance homogeneity before analysis. Statistical significance was set at *p* < 0.05.

## 3 Results

### 3.1 Single Systemic Pitolisant Injection Inhibits EtOH‐Induced Locomotor Activation, CPP, and Sedative Effect in Mice

**FIGURE 1:**
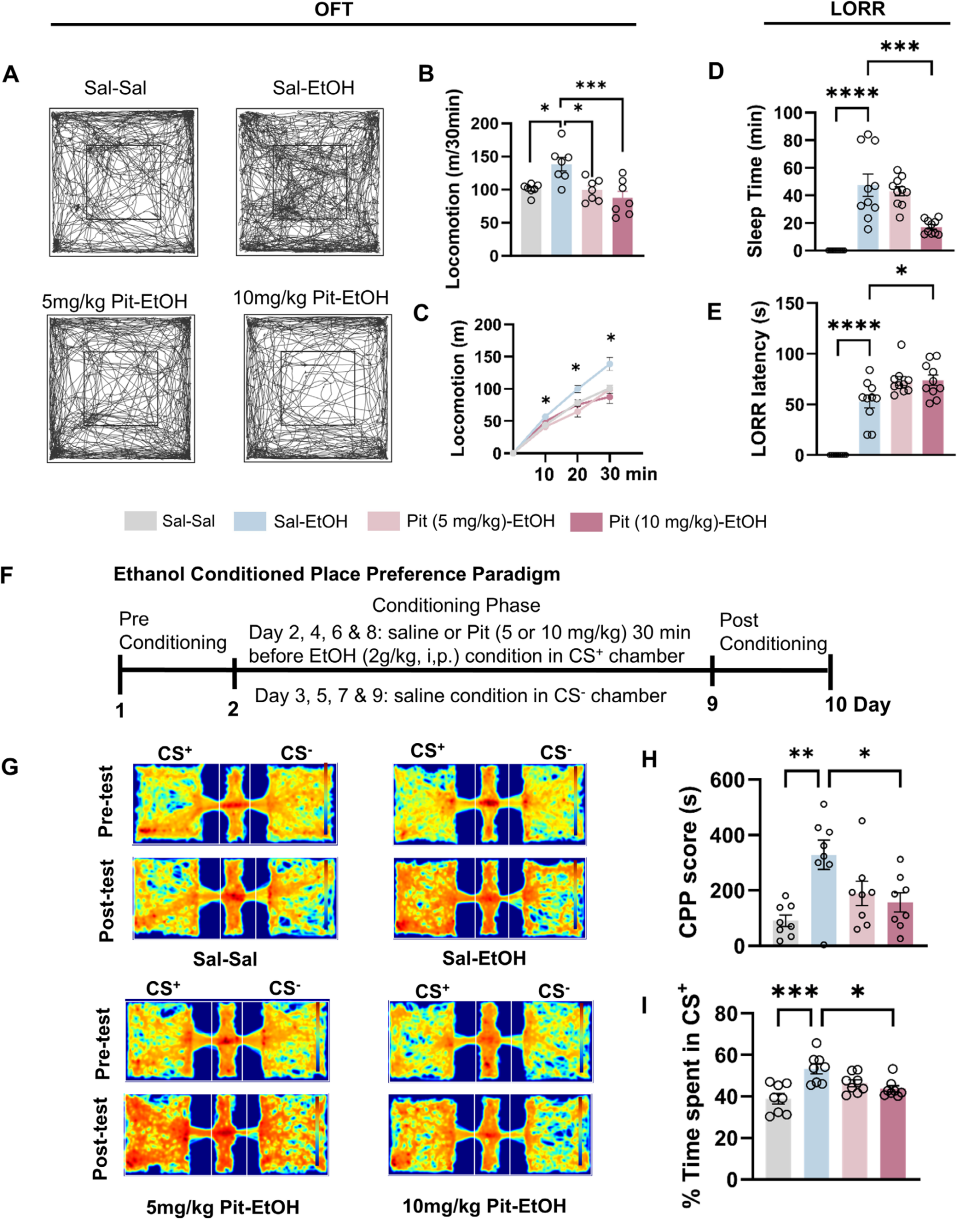
Systemic pitolisant counteracts ethanol‐induced locomotor activation, conditioned place preference (CPP), and sedation in mice. Mice received pitolisant (5 or 10 mg/kg, i.p.) or saline before the administration of ethanol (EtOH; 2 or 4 g/kg, i.p.). Locomotor activity was assessed using the open field test (OFT), the reward property was assessed by the CPP test, and sedation was evaluated via the loss of righting reflex (LORR). (A) Representative locomotor trajectories in the OFT. (B–C) Total distance traveled and 10‐min bin analysis. (D–E) LORR latency and sleep duration. F: Timeline of the CPP protocol. G: Mouse movement patterns in conditioned stimulus plus (CS+) and CS− chambers during pre‐ and post‐tests. (H–I) CPP scores and percentage of time spent in the CS+ chamber. *p < 0.05, **p < 0.01, ***p < 0.001, ****p < 0.0001, revealed by one‐way or two‐way RM ANOVA followed by Bonferroni's multiple comparisons. *n* = 8–10 mice/group for behavioral tests. All data are expressed as mean ± SEM.

Mice (*n* = 28) were first treated with pitolisant (5 or 10 mg/kg, i.p.) or saline for 30 min, followed by challenge with EtOH (2 g/kg) or an equal volume of saline. Statistical analysis revealed a significant main effect of treatment (*F*_3,24_ = 7.736, *p* = 0.0009). Acute alcohol injection was expected to significantly enhance locomotor activity compared to saline, as indicated by the greater total distance traveled (*p* = 0.0170). Notably, both 5 and 10 mg/kg of pitolisant diminished this effect, suggesting that pitolisant counteracts ethanol's locomotion‐stimulating effect (*p* < 0.05, Figure 1A–C).

For LORR test, 40 mice were divided into three groups and pretreated with pitolisant (5 or 10 mg/kg, i.p.) or saline 30 min before EtOH administration, followed by the LORR test. Statistical analysis revealed significant main treatment effects on both sleep latency (*F*_3,36_ = 53.47, *p* < 0.0001) and duration (*F*_3,36_ = 25.77, *p* < 0.0001; Figure 1D,E). EtOH (4 g/kg) induced a hypnotic state with a latency of 53.0 ± 4.68 s and a sleep duration of 47.4 ± 8.0 min. Pretreatment with 10 mg/kg pitolisant significantly delayed the latency (73.8 ± 5.32 s, *p* = 0.0364) and shortened the sleep duration (17.11 ± 1.66 min, *p* = 0.0008). In contrast, 5 mg/kg pitolisant had no significant effect on latency or duration (*p* > 0.05). These results strongly suggest that pitolisant may interfere with ethanol's hypnotic effect in a dose‐dependent manner. Additionally, as pitolisant didn't alter blood alcohol levels in EtOH‐challenged mice within 8 h post‐injection (Figure S1A), we can rule out that its effect on sedation is due to changes in alcohol metabolism.

The CPP experiment followed the timeline in Figure 1F. In the pre‐test, there was no significant difference in time spent in each chamber among 32 subjects (Figure S1B), indicating no innate chamber preference at the experiment's start. Figure 1G–I shows that after 4 cycles of pair‐conditioning, on day 10, statistical results revealed a significant treatment effect on the % Time spent in EtOH paired chamber (*F*_1,7_ = 74.90, *p* < 0.0001) and the CPP score (*F*_1,7_ = 74.90, *p* < 0.0001). Mice conditioned with EtOH spent significantly more time in the EtOH‐paired chamber than saline conditioned mice on the test day, confirming the rewarding effect of 2 g/kg EtOH [37]. Importantly, mice pretreated with 10 mg/kg pitolisant during the 4‐cycle EtOH pair‐conditioning spent significantly less time in the EtOH‐paired chamber than those in the 5 mg/kg or saline groups (*p* < 0.05). Notably, neither pitolisant dose affected total locomotor distance (Figure S1C), ruling out motor impairment as a cause of the observed effects. In addition, we did not include a pitolisant‐alone group, as previous studies showed that pitolisant has no additive property [32].

### 3.2 Single Pitolisant Injection Reduces Voluntary Alcohol Consumption of Male Mice

**FIGURE 2:**
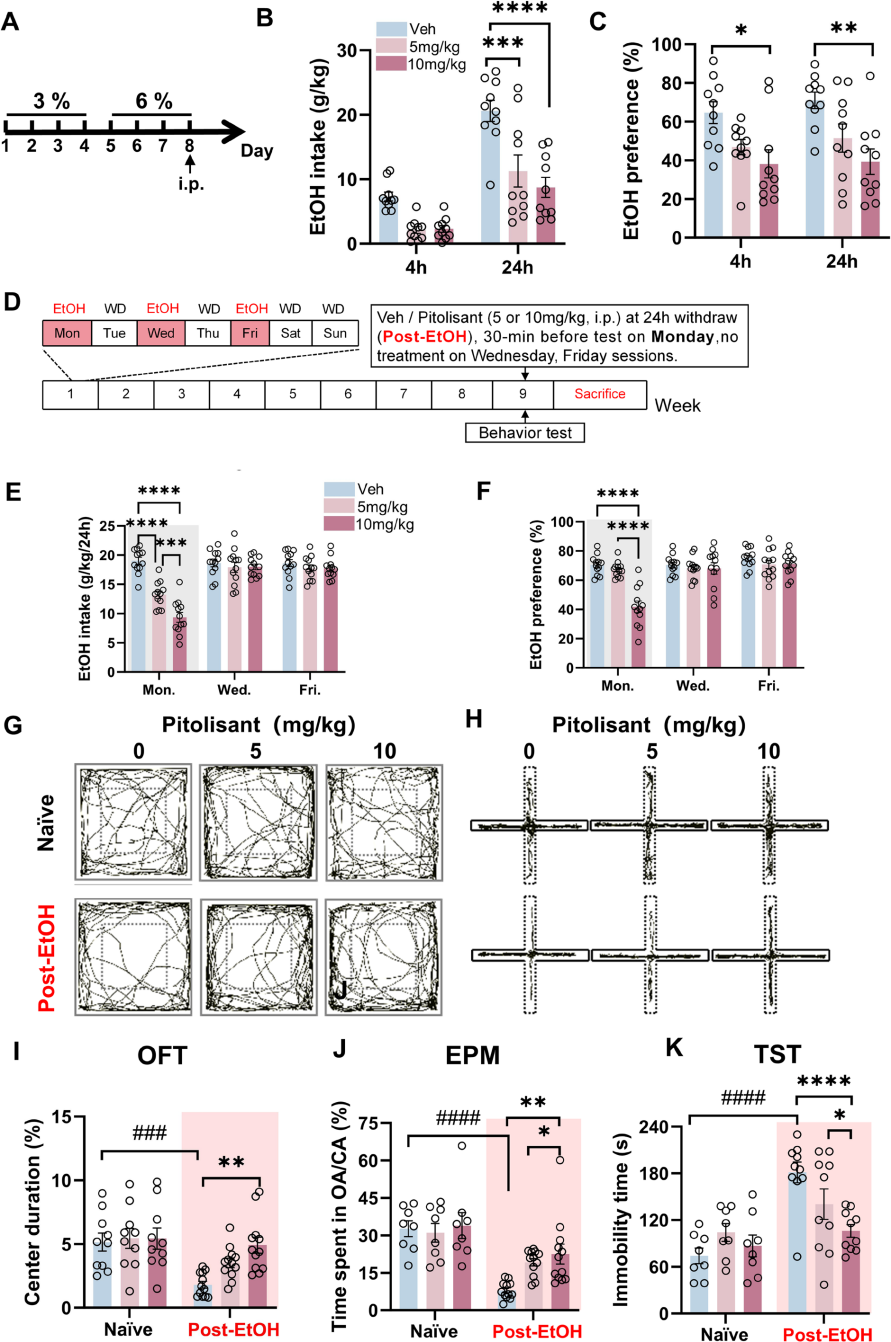
Systemic pitolisant reduces voluntary alcohol consumption and alleviates withdrawal induced anxiety and depression‐like behaviors. (A) Experimental timeline for the two‐bottle choice (2 bc) paradigms. (B–C) Ethanol intake and preference following acute pitolisant administration. (D) Timeline for chronic IA2BC paradigms and behavioral testing. (E–F) Effects of pitolisant on alcohol intake and preference during chronic exposure. (G–K) Behavioral outcomes in the open field test (OFT), elevated plus maze (EPM), and tail suspension test (TST) following withdrawal. ###*p* < 0.001 vs. Naïve, *p < 0.05, **p < 0.01 vs. ***p < 0.001 Veh within‐group, revealed by Two‐Way RM ANOVA followed by Bonferroni's post hoc test. *n* = 8–10 mice/group for behavioral tests. All data are expressed as mean ± SEM.

Next, we assess the effect of pitolisant on mice's binge drinking behavior by using a two‐bottle choice (2 bc) drinking paradigm with drug treatment design [38] (Figure 2A). On Day 8, the mice were divided into three subgroups to ensure no significant difference in average EtOH intake and preference before treatment (*p* > 0.05). During the test, one subgroup received saline while the other two were given pitolisant at 5 mg/kg and 10 mg/kg, respectively. EtOH drinking was evaluated 4 and 24 h after injection.

Statistical analysis revealed a significant main effect of treatment on intake (*F*_2,27_ = 16.91, *p* < 0.0001, Figure 2B), indicating that different pitolisant doses affected EtOH consumption. There was also a significant treatment effect on preference (*F*_2,27_ = 8.186, *p* = 0.0017, Figure 2C). Notably, a single 10 mg/kg pitolisant dose significantly reduced intake at 24 h and preference at 4 and 24 h (both *p* < 0.0001), without changing the total fluid intake (Figure S1D,E).

To explore pitolisant's effect on alcohol consumption in chronically drinking mice, we trained another group of mice (*n* = 36) to consume 20% EtOH by using IA2BC paradigm (Figure 2D) as we did before [34, 39]. During the 8‐week observation, these mice gradually increased their voluntary alcohol intake and preference [main time effects of EtOH intake (*F*_25,234_ = 2.763, *p* < 0.0001)] and preference [(*F*_25,234_ = 3.532, *p* < 0.0001)]. EtOH intake rose from 7.76 ± 0.37 to 17.40 ± 0.68 g/kg/24 h, and preference from 33.25% ± 1.72% to 66.57% ± 3.99% (Figure S2A,B). These stable levels are consistent with our previous report [34].

Subsequently, the mice were divided into three subgroups for 3 consecutive weeks, with no significant differences in intake and preference. Every Monday, 30 min before drinking, one subgroup received saline, while the other two got 5 or 10 mg/kg pitolisant. Over a 1‐week observation, statistical results showed significant main treatment effects on intake (*F*_2,33_ = 13.06, *p* < 0.0001, Figure 2E) and preference (*F*_2,33_ = 12.69, *p* < 0.0001, Figure 2F) during 24‐h sessions. *Post hoc* analysis revealed that 5 or 10 mg/kg pitolisant significantly reduced EtOH intake and preference within 24 h (*p* < 0.05), with a dose‐dependent effect. The two doses of pitolisant also increased water intake but did not alter the total fluid intake of mice (Figure S2C,D). The pharmacological effect was limited to 24‐h sessions, and the trend remained consistent over 3 weeks (Figure S2E,F).

### 3.3 Pitolisant Mitigates Withdrawal‐Induced Negative Affect in Male Mice

Thirty minutes before the behavioral tests, pitolisant or saline was administered to evaluate how pitolisant changes above behaviors. Two‐way ANOVA revealed the primary treatment significant group effects on OFT (% center duration, F_2,40_ = 4.18, *p* = 0.0224, Figure 2G,I; center entries, *F*_2,40_ = 4.343, *p* = 0.0196, Figure S3A), EPM test (% time spent in OA/CA, *F*_1,18_ = 37.83, *p* < 0.0001, Figure 2H,J; entries to OA/CA, *F*_1,18_ = 26.46, *p* < 0.0001; total entries number, *F*_2,36_ = 7.311, *p* = 0.0022, Figure S3B,C), TST (immobility time, *F*_2,32_ = 4.085, *p* = 0.0263, Figure 2K) and SPT (sucrose preference, *F*_2,36_ = 6.435, *p* = 0.0041, Figure S3D). The *post hoc* test detected that compared to the saline group, the administration of pitolisant, especially at a dose of 10 mg/kg, significantly alleviated the anxiety and depression levels in the abstinent mice (all *p* < 0.05). Notably, pitolisant did not significantly alter the locomotor activity of all the tested animals (Figure S3E).

### 3.4 Central Administration of Pitolisant Attenuates Alcohol Drinking and Abstinence‐Induced Negative Emotion

**FIGURE 3:**
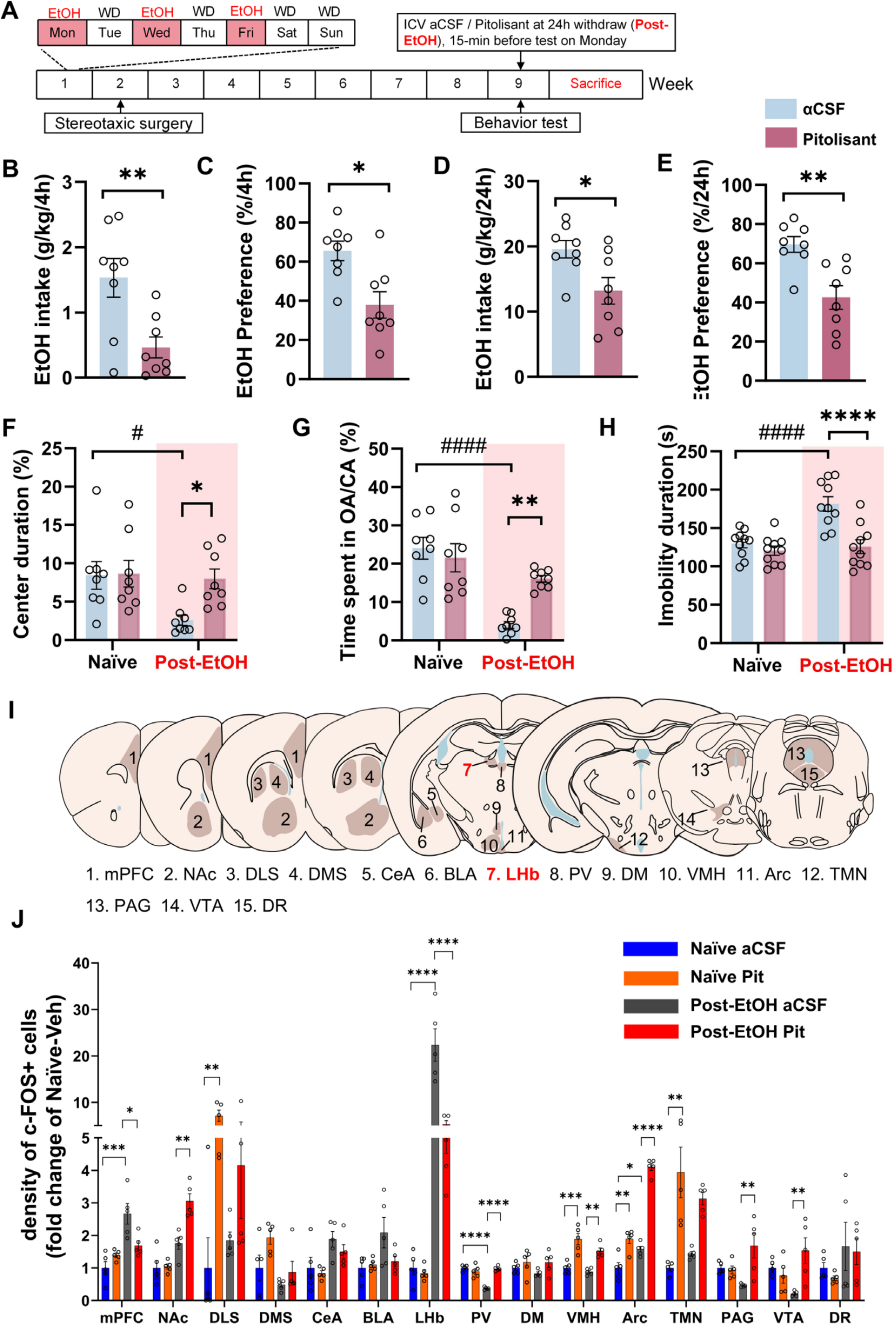
Intracerebroventricular pitolisant reduces alcohol intake, alleviates withdrawal induced negative affect, and modulates brain‐wide cFos activation. (A) Experimental timeline for ICV infusion and behavioral testing. (B–E) Ethanol intake and preference at 4 h and 24 h post‐infusion. (F–H) Behavioral outcomes in OFT, EPM, and TST. (I) Brain regions analyzed for cFos immunoreactivity. J: Fold change in cFos immunoreactive cells counting across 15 brain regions. *p < 0.05, **p < 0.01 vs. aCSF (B‐E), revealed by student t‐test; ###*p* < 0.001 vs. Naïve (F‐H), *p < 0.05, **p < 0.01 ***p < 0.001 vs. within group (J), revealed by Two‐Way ANOVA followed by Bonferroni's post hoc test. *n* = 8–10 mice/group for behavioral tests. For the histological results, *n* = 5 mice/group, each point represents the mean value of cell counting from 6 to 8 slides in each mouse. All data are expressed as mean ± SEM.

As depicted in Figure 3A, pitolisant (5 μg/200 nL/side) or an equal volume of aCSF was unilaterally microinjected into the right ventricle of both naïve and Post‐EtOH mice. Then, mood behaviors were assessed, and alcohol consumption in the Post‐EtOH group was measured in the next 24‐h session. In the Post‐EtOH mice, pitolisant significantly reduced EtOH intake and preference at 4 and 24 h post‐injection compared to aCSF microinjection (all *p* < 0.0001, Figure 3B–E), without significantly altering total fluid intake (Figure S4A–D).

A two‐way ANOVA showed significant treatment × group interaction effects on the OFT (% center duration, *F*_2,40_ = 4.183, *p* = 0.0224, Figure 3F), EPM test (% times spent in OA/CA, *F*_1,18_ = 37.83, *p* < 0.0001, Figure 3G), and TST (immobility duration, *F*_2,32_ = 4.085, *p* = 0.0263, Figure 3H). *Post hoc* tests indicated that central pitolisant treatment significantly alleviated anxiety‐like behaviors in Post‐EtOH mice compared to aCSF (all *p* < 0.05). However, they had no significant effect on the naïve control group. Pitolisant also did not affect the locomotor activity of the subjects (see detailed behavior readouts in Figure S4E–J).

Next, we evaluated the effect of central pitolisant injection on neuronal activation during 24‐h withdrawal. We compared the fold change of c‐Fos immunoreactivity (IR) cell counts between naïve mice and Post‐EtOH mice treated with either intra‐ICV infusion of pitolisant or the same volume of aCSF in 15 brain regions (Figure 3I). The selection of these regions was based on previous mouse model studies that reported brain‐wide c‐Fos activation variations due to alcohol withdrawal [40].

As shown in Figure 3J, the analysis revealed that in Post‐EtOH mice, compared to aCSF infusion, pitolisant significantly reduced the number of cFos‐IR cells in the LHb (*p* < 0.0001) and medial prefrontal cortex (mPFC, *p* < 0.05). In contrast, there was an increasing trend in cFos‐IR cell numbers in the ventral tegmental area (VTA), nucleus accumbens (NAc), arcuate nucleus (Arc), periaqueductal gray (PAG), paraventricular nucleus (PV), and ventromedial hypothalamic nucleus (VMH) (all *p* < 0.05).

Additionally, within Post‐EtOH mice, there was a significant increase in cFos‐IR cell numbers in the mPFC, LHb, and Arc. At the same time, the PV and VTA showed a decreasing trend. Also, in naïve mice, pitolisant significantly increased the number of cFos‐IR cells in the tuberomammillary nucleus (TMN), VMH, Arc, and dorsolateral striatum (DLS) compared to aCSF infusion (see detailed fold‐change in Table S3).

### 3.5 Pitolisant Modulates Post‐EtOH Induced Changes in Histaminergic Signaling and Metabolite Levels in Mouse LHb

**FIGURE 4:**
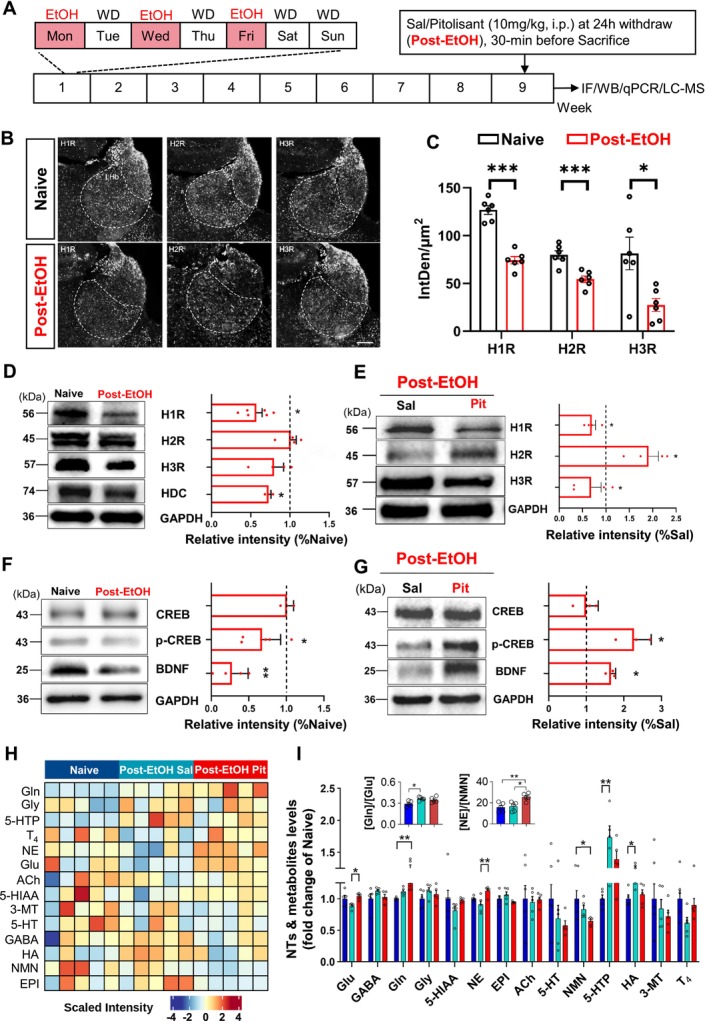
Chronic alcohol exposure alters histamine receptor expression, pCREB/BDNF signaling, and neurotransmitter levels in the LHb. (A) Experimental timeline. (B) Representative immunofluorescence images of H1R, H2R, and H3R in the LHb, scale bar = 200 μm. (C) Quantification of receptor fluorescence intensity. (D–G) Western blot analysis of H1R, H2R, H3R, HDC, pCREB, and BDNF. (H,I) Heatmap and bar graph summarizing the variations of 14 neurotransmitters and metabolites in the LHb revealed by the LC–MS test. *p < 0.05, **p < 0.01, ***p < 0.001 vs. Naïve, revealed by Unpaired t test or One‐way ANOVA followed by Bonferroni *posthoc* test, *n* = 3–5 mice/group, each molecular sample pooled from three mice. All data are shown as mean ± SEM.

We recently reported that LHb hyperactivity leads to a negative affective state [35], especially during acute alcohol withdrawal [27, 28, 30]. Inspired by an elegant single‐cell RNA sequencing study that documented the expression of *Hrh1‐3* (genes encoding Histamine Receptor H1‐3) in the mouse habenula [26], we investigated how Post‐EtOH conditions alter histamine receptor and BDNF/CREB signaling in the LHb (Figure 4A). Histological results validate the distribution of H1R, H2R, and H3R in the LHb of naïve mice. However, Post‐EtOH significantly reduced the immunofluorescence intensity of these receptors in the LHb (Figure 4B,C).

The Western blot results further revealed a significant down‐regulation of H1R, HDC, phosphorylated CREB, and BDNF protein levels in the LHb of Post‐EtOH mice compared to their naïve counterparts. Within the Post‐EtOH group, when compared to the saline control, pitolisant treatment significantly restored the reduced levels of phosphorylated CREB and BDNF, elevated H2R expression, and reduced H1R and H3R expression (Figure 4D–G).

Next, the LC–MS method was employed to measure how pitolisant changes NTs and metabolites. As shown in Figure 4H,I, among 18 metabolites, DOPA, DA, MN, and Tyramine were undetected in the LHb tissue. The three most concentrated compounds are Glu (19.05 ± 1.06 μg/g), GABA (12.83 ± 1.02 μg/g), and Gln (5.46 ± 0.056 μg/g). Conversely, the three least concentrated metabolites are T_4_ (1.40 ± 0.08 ng/g), 3‐MT (6.40 ± 1.58 ng/g), and HA (8.60 ± 0.60 ng/g).

Statistical results revealed significant differences in the Glu, Gln, NE, NMN, 5‐HTP, and HA levels in the LHb among the three groups. The *post hoc* test found that Post‐EtOH mice exhibited significantly elevated [HA] and [5‐HTP] levels compared to the naïve group (all *p* < 0.05). The Post‐EtOH condition is also featured by an increased [Gln]/[Glu] ratio, which reflects the neuronal–glial interactions and the balance of glutamatergic metabolites. Moreover, within the Post‐EtOH group, pitolisant significantly increased [NE] and [Glu] levels (all *p* < 0.05) compared to the saline control. The enhanced [NE]/[NMN] ratio indicates a relatively slow NE metabolizing rate. Furthermore, although the increase in [HA] induced by pitolisant did not reach statistical significance (*p* = 0.0524), there was a discernible upward trend in histamine levels.

### 3.6 Pharmacological Antagonizing LHb H3R Reduces Alcohol Intake and Alleviates the Alcohol Withdrawal‐Induced Negative Affective Behavior in Mice

**FIGURE 5:**
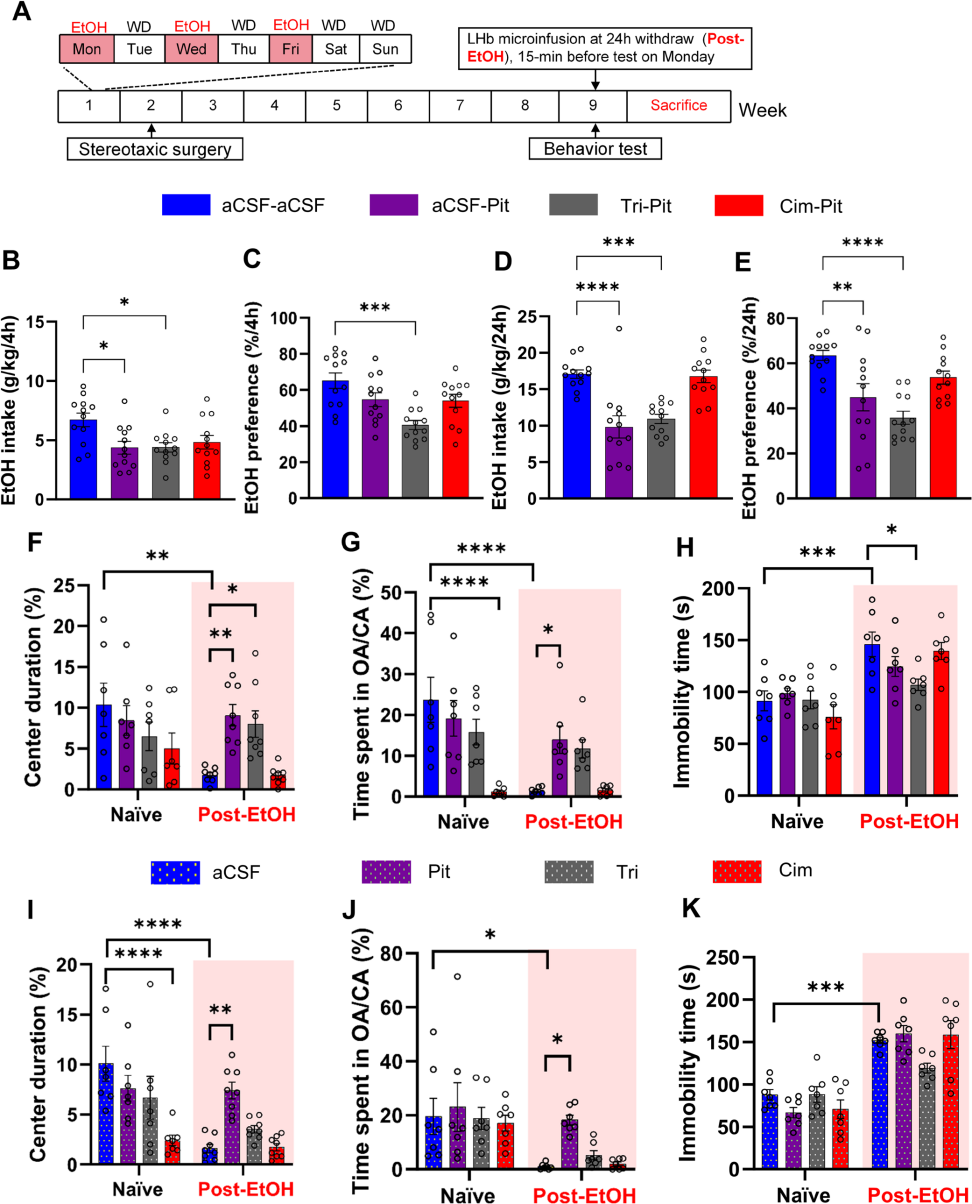
Intra‐LHb pitolisant reduces alcohol intake and withdrawal‐induced negative affect via H2R signaling. (A) Experimental timeline for intra‐LHb infusion and behavioral testing. (B–E) Ethanol intake and preference following pitolisant alone or with H1R/H2R antagonists. F–K: Behavioral outcomes in OFT, EPM, and TST across naïve and Post‐EtOH groups. *p < 0.05, **p < 0.01, ***p < 0.001 vs. Naïve+Veh, revealed by One‐way or Two‐Way ANOVA followed by Bonferroni's post hoc test. *n* = 7–12 mice/group for behavioral tests. All data are expressed as mean ± SEM.

To evaluate if pitolisant modulates EtOH consumption and mood behavior via LHb HAergic signaling, we trained an independent Post‐EtOH group of mice (*n* = 48) under the IA2BC paradigm (Figure 5A). First, we examined the effects of bilateral intra‐LHb infusion of pitolisant alone or combined with the H1R antagonist triprolidine or the H2R antagonist cimetidine on EtOH drinking and mood behaviors. The inhibitor doses were chosen based on the fact that they effectively increase brain HA levels or antagonize H1R and H2R [41].

Figure 5B–E shows significant treatment effects on EtOH intake (4 h, *F*_3,44_ = 4.490, *p* = 0.0078; 24 h, *F*_3,44_ = 15.37, *p* < 0.0001) and preference (4 h, *F*_3,44_ = 7.794, *p* = 0.0003; 24 h, *F*_3,44_ = 9.630, *p* < 0.0001) at the two time points. *Post hoc* test revealed that pitolisant significantly reduced 24‐h EtOH intake and preference compared to vehicle injection (*p* < 0.05), without affecting total fluid intake. The alcohol drinking suppression by intra‐LHb pitolisant was reversed by H2R antagonist cimetidine, not by H1R antagonist triprolidine. Intra‐LHb triprolidine Figure S5 or cimetidine microinjection alone did not significantly alter alcohol drinking.

Regarding abstinence‐induced negative affect, statistical analysis revealed a significant group × treatment interaction effect on the OFT (center duration, *F*_3,52_ = 6.008, *p* = 0.0014), a significant group effect on the EPM test (time spent in OA/CA, *F*_1,48_ = 17.33, *p* = 0.0001), and a group × treatment effect on TST (immobility duration, *F*_3,48_ = 4.673, *p* = 0.0061) suggesting different treatments altered anxiety‐ and depression‐like behavior. In the Post‐EtOH group, pitolisant attenuated anxiety‐like behavior (all *p* < 0.05). This effect was absent in groups treated with pitolisant + H2R antagonist cimetidine or cimetidine alone compared to the vehicle group (Figure 5F–H) and Figure S6. In the Naïve group, pitolisant did not significantly change anxiety‐ or depression‐like behavior. However, pitolisant + H2R antagonist cimetidine significantly reduced time in OA/CA (*p* < 0.05). Intra‐LHb triprolidine alone also did not significantly change these behaviors in either group (Figure 5I–K, Figure S7).

## 4 Discussion

### 4.1 Summary of Main Results

This study demonstrates that pitolisant, an FDA‐approved H3R antagonist, effectively suppresses EtOH‐induced locomotor stimulation and reward, reduces voluntary alcohol consumption, and alleviates withdrawal‐induced anxiety and depression‐like behaviors in mice. These effects appear to be mediated through histaminergic signaling in the LHb, a brain region increasingly recognized for its role in mood regulation and addiction.

### 4.2 Pitolisant's Effects on Alcohol Reward, Sedation, and Withdrawal in AUD

AUD involves a transition from positive to negative reinforcement [42], driven by changes in dopaminergic signaling. EtOH enhances dopamine release in the VTA and NAc [43, 44], reinforcing consumption [45]. Over time, this reward circuitry shifts toward the dorsolateral striatum, promoting compulsive drinking [46]. Our findings show that pitolisant disrupts EtOH‐induced CPP and locomotor activation, align with prior studies reporting that H3R antagonists (e.g., DL77, ST1283, ciproxifan) reduce alcohol reward and intake [11, 13]. These results infer that H3R or endogenous HA level in the dopaminergic reward circuitry regulates the rewarding property of alcohol.

Histamine's role in modulating dopamine release is complex. First, antagonists like pitolisant may inhibit mesolimbic DA function. This is because H3R antagonists affect histaminergic autoreceptors more than heteroreceptors beyond TMN [47]. The nucleus accumbens and dorsal striatum receive weak histaminergic innervation but high presynaptic heteroreceptor H3R densities [48]. Antagonizing these receptors does not increase DA release as expected but rather inhibits DA release [49] by activating H3Rs at terminals via an increase in brain histamine levels. Secondly, elevated histamine may inhibit DA‐mediated behavior via postsynaptic H1R and H2R signaling, since D1R‐H3R heterodimerization in the medium spiny neurons of the striatum switches the D1 dopamine receptor signaling from Gs/olf to Gi [46]. This mechanism may explain pitolisant's ability to blunt EtOH reward and reduce alcohol‐seeking behavior. Furthermore, the enhanced GABAergic release in the substantia nigra by pitolisant [46] may inhibit dopaminergic cell firing and prevent the transition from initial casual drinking to habitual drinking.

Pitolisant also mitigates the sedative and hypnotic effects of high‐dose EtOH. It likely achieves this by restoring the excitability of histaminergic neurons in the TMN. The excitability of these neurons was previously suppressed due to EtOH‐potentiated GABAergic transmission [50]. This finding supports the hypothesis that histamine can counteract EtOH‐induced sedation.

Beyond reward modulation, pitolisant alleviates withdrawal‐induced negative affect. Chronic alcohol exposure, characterized by repeated cycles of excessive drinking and withdrawal in mice, disrupts the neurotransmitter balance and stress‐related peptide levels in the brain during abstinence [43]. As a consequence, the mice develop negative affective states and behaviors such as anxiety and depression, which is consistent with recent reports [34] and supports the view that negative reinforcers promote drinking relapse and the development of AUD.

### 4.3 Pitolisant Normalizes Neural Changes in Alcohol Withdrawn Mice's LHb

We found region‐specific neuronal activation changes in the brains of abstinent mice during withdrawal, mainly characterized by increased cFos‐IR cell numbers in the LHb, mPFC, PV, and Arc, and decreased numbers in the VTA. These regions are crucial for emotion regulation in AUD, highlighting the interplay of neural systems [40]. Aberrant activation in the LHb, partly through the hyperactive LHb‐RMTg circuitry [29], drives monoamine hypofunction and the reward deficiency state, which are closely linked to anxiety‐and depression‐like behaviors associated with alcohol withdrawal [27, 28, 30]. Pitolisant reversed these changes, especially in the LHb, suggesting it normalizes hyperactivity in mood‐related circuits.

We confirmed the presence of histamine receptors (H1R, H2R, H3R) in the LHb. Histological and molecular analyses revealed that alcohol withdrawal downregulated H1R, H3R, and HDC in this area, suggesting that alcohol disrupts histamine synthesis and turnover, as reported in a previous study [51]. The above interpretation is supported by current quantitative LC–MS results showing that the LHb of Post‐EtOH mice is characterized by an elevated histamine level. This finding aligns with a canonical study reporting elevated brain histamine in an alcohol‐preferring rat line [52]. Moreover, systemic pitolisant administration elicited a further increasing trend in histamine level, even though the endogenous HA level was ~2000‐fold lower than that of primary neurotransmitters like glutamate. This result further indicates that the histaminergic signaling modulated by H3R is involved in the LHb neuroadaptation in response to chronic alcohol exposure.

We also found that EtOH exposure decreased brain‐derived neurotrophic factor (BDNF) levels and cAMP‐response element‐binding protein (CREB) phosphorylation in the habenula. The current result was in agreement with a report that BDNF treatment alleviated alcohol‐withdrawal anxiety in mice and normalized neurons [53], and another report that the habenula BDNF/TrkB/CREB cascade was reduced and normalized by acupuncture using a rat depression model [54], suggesting endogenous BDNF signaling stabilizes mood via neuroplasticity [55]. Notably, intra‐LHb infusion of pitolisant replicated systemic effects, and co‐administration with the H2R antagonist cimetidine blocked its benefits, implicating H2R signaling in pitolisant's mechanism.

Moreover, strong evidence suggests that LHb H3R functions as heteroreceptors, modulating other neurotransmitters beyond the HA system. Our LC–MS data revealed Post‐EtOH features increased 5‐HTP and the [Gln]/[Glu] ratio in the LHb. We previously reported elevated 5‐HT release in the LHb of Post‐EtOH rats. This increased 5‐HT release activated the 5‐HT2C receptor, which suppressed the M‐channel and drove neuronal hyperactivity and negative affect during withdrawal [27]. Additionally, AUD patients demonstrate a higher brain [Gln]/[Glu] ratio [56], which is functionally linked to the increased impulsivity in adults with a family history of alcoholism [57].

More importantly, pitolisant significantly increased the [NE]/[NMN] ratio in the LHb of Post‐EtOH mice, suggesting that it may limit the metabolic rate of NE. Our results seem to conflict with a recent study [58] that emphasized phasic, rather than tonic, NE release is required to influence the LHb neuron–glia interaction, driving LHb neuronal hyperactivity and depression in a positive feedback loop. However, it remains to be elucidated whether pitolisant‐induced NE release occurs in a slow ramping tonic or rapid phasic burst manner. Tonic NE release is the primary neural mechanism underlying antidepressants such as NRIs and SSRIs.

One limitation of this study is the exclusive use of male mice. Estrogen can regulate the brain H1R in females [59], which complicates the interpretation of the data. As a result, to ensure the reliability and consistency of our experimental results, female mice were excluded from the study. Future research should incorporate female subjects. This is necessary to explore the sex‐specific aspects of this mechanism and improve the generalizability of our findings.

In conclusion, pitolisant shows promise as a novel treatment for AUD by targeting H3R in the LHb to reduce alcohol consumption and withdrawal‐induced negative affect. This study provides mechanistic insights into histaminergic regulation of addiction and supports further exploration of pitolisant's clinical potential.

## Author Contributions

Conceptualization: Rao Fu and Yan Zhao; methodology: Yan Zhao, Yixin Fu, Bingqing Chen, Zanhao Yang, Hui Qi, Duo Chen, Xiaojiao Han, Lipeng Zho, and Ying Tang; visualization: Yixin Fu, Tianhao Liu, Zanhao Yang, Zhengzhong Yang, Juntao Yang; resources: Rao Fu and Yan Zhao; data interpretation: Yixin Fu, Yan Zhao, and Rao Fu; writing‐original draft: Yixin Fu, Yan Zhao, Tianhao Liu; writing‐review and editing: Rao Fu, Chao‐Yu Miao, and Jianghong Ye; project administration: Rao Fu; funding acquisition: Yan Zhao and Rao Fu. All authors have read and agreed to the published version of the manuscript.

## Funding

This work was supported by National Natural Science Foundation of China, 82071496 to Rao Fu, 82571703 to Rao Fu. Natural Science Foundation of Guangdong Province, 2024A1515013166 to Rao Fu Shenzhen Science and Technology Innovation Program, JCYJ20220530145401003 to Rao Fu. Fund of Shenzhen Key Laboratory, ZDSYS20220606100803007. Clinical Research Special Program of the Shanghai Municipal Health Commission, 202140011 to Yan Zhao.

## Ethics Statement

The animal experiments were approved by the Sun Yat‐sen University Animal Experimentation Ethics Committee (Approval No. SYXK 2017–0081).

## Conflicts of Interest

The authors declare no conflicts of interest.

## Supporting information


**Data S1:** Supporting Figures and Tables.

## Data Availability

Data are provided within the manuscript or Supporting Information files.
