# Supplementary material for: Pitolisant Inhibits Alcohol Drinking and Improves Withdrawal Negative Affect Through Lateral Habenula Histaminergic Signaling in Mice
Source: CNS Neurosci Ther. 2026 Jan 6;32(1):e70732. doi: 10.1002/cns.70732 (PMC12771655; doi:10.1002/cns.70732)
Supplement: Supplementary file 1 — Data S1: Supporting Figures and Tables. [file CNS-32-e70732-s001.docx]

SUPPLEMENTARY METHODS AND MATERIALS

1. **Methods and materials**

**1.1 Behavioral tests**

*Loss of righting reflex (LORR)*

The loss of righting reflex (LORR) test, based on established methods[1], was used to evaluate pitolisant’s effect on ethanol-induced sedation. Mice were intraperitoneally (i.p.) injected with either pitolisant or saline. Thirty minutes later, they received an i.p. ethanol injection at 4 g/kg (20% v/v). Immediately after the ethanol injection, each mouse was placed on its back on a flat surface to assess LORR. LORR was defined as the mouse’s failure to right itself within 30 seconds in three consecutive attempts. The LORR latency was the time from ethanol injection to the first failure. Sleep time was until the mouse could right itself thrice within 30 seconds.

*Ethanol induced locomotion activation*

The open field apparatus, made of dark blue polypropylene plastic and placed on the floor, consisted of a 0.5 m×0.5 m×0.5 m square acrylic plastic chamber. Each animal was tested for 20 minutes, and the apparatus was cleaned with 70% diluted ethanol between trials. The total distance traveled by the animal was recorded.

At the start of the experiment, the experimenter gently took a mouse from its home cage and placed it in a corner of the open field. The mouse was then allowed to freely explore for 5 minutes, during which its movement was recorded, tracked, and analyzed using the BAS-100 animal behavior analysis system. After the test, the mouse was returned to its home cage, and the apparatus was cleaned with 75% ethanol to remove feces, urine, and odor, avoiding interference for the next test.

Recorded parameters included the number of entries and time percentage in the center and peripheral areas, total distance traveled, and the trajectory map. The percentage of time in the center and the number of center entries were used to measure anxiety, while the total distance traveled assessed locomotor ability as we previously described[2].

*The expression of ethanol-induced conditioning place preference*

The ethanol-induced conditioned place preference test was conducted to evaluate the effect of pitolisant on the reinforcing effect of ethanol. The CPP apparatus (CPP-100, Taimeng, China) consisted of two equal-sized compartments (20cm×20cm×15cm) and one gray compartment (6cm×20cm×15cm). The two equally sized compartments were equipped with different visual and tactile cues. One was a black wall with a gridded floor, while the other was a black/white striped wall with a round hole on the floor. The time spent by the mice in each compartment and the distance travelled were recorded using an infrared monitoring system.

The CPP procedure was conducted as a well-established procedure[3], consisting of three phases over 10 consecutive days.

Pre-conditioning (Day 1): The mouse was administered saline (12.5 ml/kg, i.p.) and immediately placed in the apparatus with a smooth paper floor. The mice had access to the entire apparatus for 30 min.

Conditioning phase (Day 2-9): Starting the day after pre-conditioning, experiments consisted of eight 5-min sessions (4 saline and 4 ethanol pairings). The approximately equal numbers of mice were randomly assigned to the Grid and Hole subgroups based

on the CS^+^ floor type. On each 30-minute conditioning trial, the apparatus contained only one floor type,

CS^+^ trials: Grid or Hole subgroup mice were i.p. injected with ethanol [2 g/kg, 20% (v/v), i.p.] immediately before being placed in the chamber with the corresponding floor (grid or hole). We chose this 2 g/kg dose as it induces intermediate place conditioning in C57BL/6J mice, which allows us to observe how pitolisant may either increase or decrease the ethanol-induced CPP.

Each subgroup was exposed to the opposite floor type during the CS- trials immediately after saline injection. Four trials of each type were given on alternating days in a counterbalanced order to equalize exposure to floor types, ethanol, and saline. Before confinement to the ‘conditioned’ compartment, mice received two i.p. injections (30 min apart) in one of three groups: vehicle and saline (Veh-Sal), vehicle and ethanol (Veh-EtOH), or pitolisant and ethanol (pitolisant-EtOH).

Post-conditioning (Day 10): On day 10, mice were allowed to access the whole apparatus for 30 minutes, and the time in each chamber was recorded. The preference score was defined as the difference in time spent in the ethanol-paired chamber on the post-conditioning day vs the pre-conditioning day. Positive or negative scores indicated preference or aversion, respectively.

*Open Field Test*

The procedure followed our previously described methods[4]. Each mouse was placed in the center of an open field apparatus (50 × 50 × 50 cm) and recorded for 5 minutes. We measured: (1) entries and time spent in the central zone (25 × 25 cm), and (2) total distance traveled. Movement distance was used to assess locomotor activity, while time in the central zone evaluated anxiety-like behavior. Between trials, the apparatus was cleaned with isopropanol solution to eliminate odor cues from previous subjects.

*Elevated plus maze*

The procedure followed our previously described methods[2,4]. The elevated plus maze was made out of black polypropylene and was raised 50 cm. It consisted of two arms (65cm×6cm) arranged in a plus configuration and intersecting with a central platform. The open arms had a 1-cm border around their perimeter, and the closed arms had a 20-cm translucent wall. A mouse was placed in the center of the apparatus facing an enclosed arm, and every 10-minute session was videotaped. The apparatus was cleaned with diluted isopropyl alcohol between animals. The number of entries into the open arm and the time spent in the open arm were scored. The total number of entries into the arms (enclosed plus open) was also calculated.

*Tail suspension test*

The procedure followed our previously described methods[2]. The testing setup comprised white acrylic walls (measuring 20 cm × 40 cm × 60 cm), with one side left open to facilitate video recording of the animals' activities. The center of the apparatus was illuminated with a brightness of 250 lx. The configuration allowed for the concurrent testing of two animals, and their separation was achievable by inserting an opaque partition at the center of the apparatus. Each mouse was suspended by its tail approximately 60 cm above the chamber floor using adhesive tape positioned less than 1 cm from the tail's tip. A video camera recorded the resulting behaviors for 6 minutes. Two experimenters, blind to the animal treatment history, later analyzed the videos and scored the count of immobility episodes and the overall duration of immobility using a

time-sampling technique every 2 seconds of the 5-minute test. The cumulative duration of time during which each mouse remained immobile was quantified.

*Sucrose preference test*

The sucrose preference test was used to assess anhedonia-like behavior of mice, as we previously reported[2,4]. Briefly, SPT was conducted 24 h after ethanol withdrawal. Before the test, mice were deprived of food and water for 20 h, after which they received a bottle of 1% sucrose solution and a bottle of water for 4 h. Sucrose intake was calculated based on the consumption of milligrams of sucrose per gram of body weight. The preference for sucrose was calculated based on the proportion of consumed sucrose solution in the overall amount of drinking liquid.

**1.2 Chemicals and application**

All the chemicals used in the current study below were analytical grade and purchased from Aladdin (China): sodium chloride (NaCl), dibasic sodium phosphate (Na_2_HPO_4_), sodium dihydrogen phosphate (NaH_2_PO_4_), TritonX-100, polyformaldehyde, sucrose.

**1.3 Immunofluorescence**

Immunofluorescence (IF) was processed as in previous reports to evaluate the brain cFos and histamine receptors expression in the neurons[5,6]. Briefly, brain sections were incubated using primary antibodies against cFos rabbit mAb (1:3000, #2250, Cell Signaling Technology, Inc.), HRH1 rabbit mAb (1:1000, KleanAB, Inc.), HRH2 rabbit mAb (1:1000, KleanAB, Inc.), and HRH3 rabbit mAb (1:1000, KleanAB, Inc.) at 4 °C overnight. After rinsing in 0.01 M PBS, the sections were incubated with goat anti-rabbit IgG (H+L), F(ab')2 Fragment (Alexa Fluor® 555 Conjugate) (1:2000, Cell Signaling Technology, Inc.) for 2 hours in the dark. Finally, after three washes in PBS, sections were mounted on glass slides using an antifade mounting medium and examined under an Olympus BX63 fluorescence microscope (Olympus, Japan). Sections from each experimental group were processed simultaneously. The average value across all sections for each animal was then determined.

The c-Fos-IR cell number and immunofluorescence intensity analysis was conducted as we reported[6]. Briefly, two investigators blinded to the treatment history counted the number of immunopositive cells of each image (10× images of the interested region). For a neuron to be considered c-Fos-IR, the nucleus must be stained with a characteristic round red fluorescence dot confirmed by DAPI counterstaining. Counts were determined for each hemisphere individually, and an average value for both hemispheres of each section was calculated. We counted the number of IR cells per level in each rat for 5-6 sections and averaged the results. The average value across all sections for each animal was then determined[6].

**1.4 Western blot analysis**

After the final behavioral test, the mouse was sacrificed under deep anesthesia with isoflurane. Then, transcardially ice-cold saline perfusion was performed, and the mouse was decapitated for rapid brain dissection. Subsequently, the mouse brain was cut with a vibratome into ice-cold artificial cerebral fluid (aCSF) containing (in mM): 126 NaCl, 2.5 KCl, 1.25 NaH₂PO₄, 1 MgCl₂, 2 CaCl₂, 25 NaHCO₃, 1 L-ascorbate, and 11 glucose, and saturated with 95% O₂/5% CO₂ (carbogen).

Tissue containing the LHb was harvested from three to four 400-μm-thick coronal slices on ice by punching them out with a stainless steel cannula (Brain Punch Set, #57401, Stoelting Co., IL, USA). Tissue harvested from 3 mice was pooled and served as each sample in the molecular experiments.

*Western blotting assay*: For whole lysate protein isolation, mechanically pulverized LHb was resuspended in RIPA buffer (Thermo Fisher Scientific) and briefly homogenized with a handheld sonicator. Protein concentration was determined using the BCA protein assay (Pierce Biotechnology, Rockford, IL, USA). For each sample, 40 μg of protein extracts were denatured and subjected to 4-15% Tris–HCl precast Mini Protean® TGX™ gels (Bio-Rad, Philadelphia, PA) for electrophoresis. Protein-separated PVDF membranes (Bio-Rad) were blocked with 5% non-fat milk (Bio-Rad) in TBST (in mM, 24 Tris, pH 7.4, 137 NaCl, 2.7 KCl, and 0.05% Tween 20) for 2 h at room temperature and incubated with primary antibodies (Please see Table 2 for antibodies information) overnight at 4 °C. After washing with TBST, the membranes were incubated for 2 h with IgG-HRP conjugated antibody (1:2500, Jackson Immuno-Research, West Grove, PA) at room temperature and developed with ECL solutions (PerkinElmer, Waltham, MA). All protein signals were analyzed by scanning densitometry using Image Lab software (Bio-Rad) and normalized to GAPDH. All experiments were repeated three times with similar results. The data presented represent the results of one of the triplicate experiments.

**1.5 LC-MS analysis for Neurotransmitters and metabolites**

For sample preparation, we added 500 μL of pre-chilled (4 °C) acetonitrile-isopropanol-water (3:3:2, v/v/v) to every 10 mg of frozen brain tissue. We homogenized the mixture on ice for 2 minutes using a Bioruptor sonicator (Diagenode), then centrifuged it at 13,000g and 4 °C for 15 minutes. The supernatant was dried at room temperature, and the residue was re-dissolved in 100 μL of methanol-water (1:1, v/v). After a final 15-minute centrifugation, the supernatant was collected into vials for analysis.

Next, we performed LC/MS analysis following the method we recently reported[7]. We used a Shimadzu Nexera X2 LC-30AD system coupled with a Phenomenex Kinetex F5 column (2.6 μm, 3 × 100 mm) and a triple quadrupole mass spectrometer (QTRAP 5500, AB SCIEX) for the analysis. The 18 metabolites [include glycine, GABA, histamine, tyramine, acetylcholine, glutamine, glutamate, dopamine, 3-methoxytyramine(3-MT), norepinephrine, serotonin, epinephrine, metanephrine (MN), 5-Hydroxyindole-3-acetic acid, DOPA, 5-hydroxy-L-tryptophan (5-HTP), thyroxine, normetanephrine (NMN)] were detected in both electrospray negative-ionization and positive-ionization modes.

Samples with an injection volume of 2 μL were loaded onto the 40°C maintained column via the LC autosampler, with a mobile phase flow rate of 200 μL/min. The gradient elution program used 0.1% formic acid in water (solvent A) and 100% acetonitrile (solvent B). It started with 100% A for 2.5 min, then linearly decreased to 70% A over 9 min, and further decreased to 0% A over 1 min. The 0% A condition was held for 5.4 min, followed by a rapid return to the initial 100% A in 0.1 min and a 2.5-min re-equilibration.

To ensure reproducibility, we analyzed pooled quality control samples every eighth injection during the analytical sequence. We quantified widely targeted metabolites using multiple reaction monitoring (MRM) mode and optimized the MRM transitions for metabolites in the MT1000 standard library (Shanghai BioProfile) with compound-specific declustering potentials and collision energies.

The mass spectrometry parameters were standardized as follows: an ion source temperature of 550°C, a nebulizer gas (Gas1) pressure of 40 psi, a heater gas (Gas2) pressure of 50 psi, a curtain gas pressure of 35 psi, and ion spray voltages of -4500 V in negative-ionization mode and +5500 V in positive-ionization mode.

**Table S1 Summary of animal numbers in each experiment**

| Group | Subgroup | Treatment or manipulation | Test |  |
| --- | --- | --- | --- | --- |
| 1 | Sal-Sal n = 7  Sal-EtOH n =7  Pit (5 mg/kg)-EtOH n = 7  Pit (10 mg/kg)-EtOH n = 7 | Pitolisant (5 or 10 mg/kg, i.p.) EtOH injection (2 g/kg, i.p.) | Locomotion  (Fig. 1. A-C) |  |
|  |  |  |  |  |
| 2 | | Sal-Sal n = 10  Sal-EtOH n =10  Pit (5 mg/kg)-EtOH n = 10  Pit (10 mg/kg)-EtOH n = 10 | Pitolisant (5 or 10 mg/kg, i.p.) EtOH injection (4 g/kg, i.p.) | LORR (Fig. 1. D-E) |
|  | |  |  |  |
| 3 | Sal-Sal n = 8  Sal-EtOH n =8  Pit (5 mg/kg)-EtOH n = 8  Pit (10 mg/kg)-EtOH n = 8 | Four times injections of  Pitolisant (5 or 10 mg/kg, i.p.) EtOH injection (2 g/kg, i.p.) | CPP test (Fig. 1. F-I) |  |
|  |  |  |  |  |
| 4 | Sal n =10  Pit (5 mg/kg) n = 10  Pit (10 mg/kg) n = 10 | 2BC drinking training  Pitolisant (5 or 10 mg/kg, i.p.) | EtOH consumption  (Fig. 2. A-C) |  |
|  |  |  |  |  |
| 5 | Sal n =12  Pit (5 mg/kg) n = 12  Pit (10 mg/kg) n = 12 | IA2BC drinking training  Pitolisant (5 or 10 mg/kg, i.p.) | EtOH consumption  Negative affect  (Fig. 2. D-K) |  |
|  |  |  |  |  |
| 6 | Naïve aCSF n = 8  Naïve Pit n = 8  Post-EtOH aCSF n = 8  Post-EtOH Pit n = 8 | IA2BC drinking training  Pitolisant (5μg, i.c.v.)  aCSF (200nl/side i.c.v.) | EtOH consumption  Negative affect  c-Fos cells counting  (Fig. 3) |  |
|  |  |  |  |  |
| 7 | Naïve n = 24  Post-EtOH n = 24  Naïve n = 15  Post-EtOH n = 30 | IA2BC drinking training | Immunofluorescence  qPCR, WB, LC-MS (Fig. 4) |  |
|  |  |  |  |  |
| 8 | Naïve  aCSF- aCSF n =12  aCSF-Pit n =12  Tri-Pit n=12  Cim-Pit n=12  Post-EtOH  aCSF- aCSF n =12  aCSF-Pit n =12  Tri-Pit n=12  Cim-Pit n=12 | IA2BC drinking training  Pitolisant (5μg, LHb)  aCSF (200nl/side LHb) | EtOH consumption  Negative affect  (Fig. 5) |  |

**Table S2 The antibodies used in this study**

| **Antibody** | **Species/Clonality** | **Source (Catalog No.)** | **Dilution** | **Usage** |
| --- | --- | --- | --- | --- |
| **Primary Antibodies** |  |  |  |  |
| HRH1 polyclonal | Rabbit/ Monoclonal | KleanAB | 1:1000 | WB/IF |
| HRH2 polyclonal | Rabbit/ Monoclonal | KleanAB | 1:1000 | WB/IF |
| HRH3 polyclonal | Rabbit/Monoclonal | KleanAB | 1:1000 | WB/IF |
| HDC polyclonal | Rabbit/ Monoclonal | KleanAB | 1:1000 | WB/IF |
| c-Fos | Rabbit/Monoclonal | CST | 1:1000 | IF |
| **Secondary Antibodies** |  |  |  |  |
| HRP conjugated antibody | goat anti-rabbit IgG | CST (7074) | 1:10000 | WB |
| Alexa Fluor^®^ 555 Conjugate Secondary Antibody | Anti-rabbit IgG (H+L), F(ab')2 Fragment | CST (4413) | 1:2000 | IF |
| Alexa Fluor^®^ 488 Conjugate Secondary Antibody | Anti-mouse IgG (H+L), F(ab')2 Fragment | CST (4408) | 1:2000 | IF |

WB: Western Blotting, IF: Immunofluorescence

SUPPLEMENTARY RESULTS

**Table S3. Fold Change of c-Fos Immunoreactive Cell Counts in Naïve and Post-EtOH Mice with or without Central Pitolisant Administration**

| Brain  Region | Naïve:Veh  vs  Naïve:  Pitolisant | Naïve:Veh  vs  Post-EtOH:  Veh | Naïve:Veh  vs  Post-EtOH:  Pitolisant | Naïve:Pitolisant  vs  Post-EtOH:  Veh | Naïve:Pitolisant  vs  Post-EtOH: Pitolisant | Post-EtOH:Veh  vs  Post-EtOH Pitolisant | Column Factor |
| --- | --- | --- | --- | --- | --- | --- | --- |
| mPFC | 0.7244 | 0.0002 | 0.1623 | 0.0025 | 0.8959 | 0.0222 | F (1, 16) = 2.066 |
| NAc | >0.9999 | 0.0727 | <0.0001 | 0.0994 | <0.0001 | 0.0012 | F (1, 16) = 12.21 |
| DLS | 0.0082 | 0.996 | 0.3275 | 0.0251 | 0.3912 | 0.6592 | F (1, 16) = 14.16 |
| DMS | 0.1713 | 0.7593 | 0.9997 | 0.0123 | 0.0915 | 0.921 | F (1, 16) = 5.582 |
| TMN | 0.0006 | 0.9753 | 0.0107 | 0.0027 | 0.6902 | 0.0515 | F (1, 16) = 33.09 |
| CeA | 0.9975 | 0.0986 | 0.6252 | 0.0369 | 0.3241 | 0.8449 | F (1, 16) = 1.341 |
| BLA | >0.9999 | 0.0603 | 0.9959 | 0.103 | >0.9999 | 0.1706 | F (1, 16) = 2.207 |
| LHb | >0.9999 | <0.0001 | 0.4997 | <0.0001 | 0.4596 | <0.0001 | F (1, 16) = 22.96 |
| PV | 0.6229 | <0.0001 | 0.9997 | <0.0001 | 0.8275 | <0.0001 | F (1, 16) = 24.94 |
| DMH | 0.9067 | 0.9281 | 0.9154 | 0.3407 | >0.9999 | 0.3533 | F (1, 16) = 4.361 |
| DLH | 0.0001 | 0.8737 | 0.0167 | <0.0001 | 0.1221 | 0.0036 | F (1, 16) = 49.64 |
| Arc | 0.001 | 0.038 | <0.0001 | 0.477 | <0.0001 | <0.0001 | F (1, 16) = 173.9 |
| PAG | >0.9999 | 0.3908 | 0.1669 | 0.5235 | 0.1116 | 0.0036 | F (1, 16) = 8.173 |
| VTA | 0.9824 | 0.1693 | 0.5654 | 0.5189 | 0.1923 | 0.0066 | F (1, 16) = 5.332 |
| DR | 0.9962 | 0.8741 | 0.9623 | 0.5518 | 0.7266 | >0.9999 | F (1, 16) = 0.3137 |


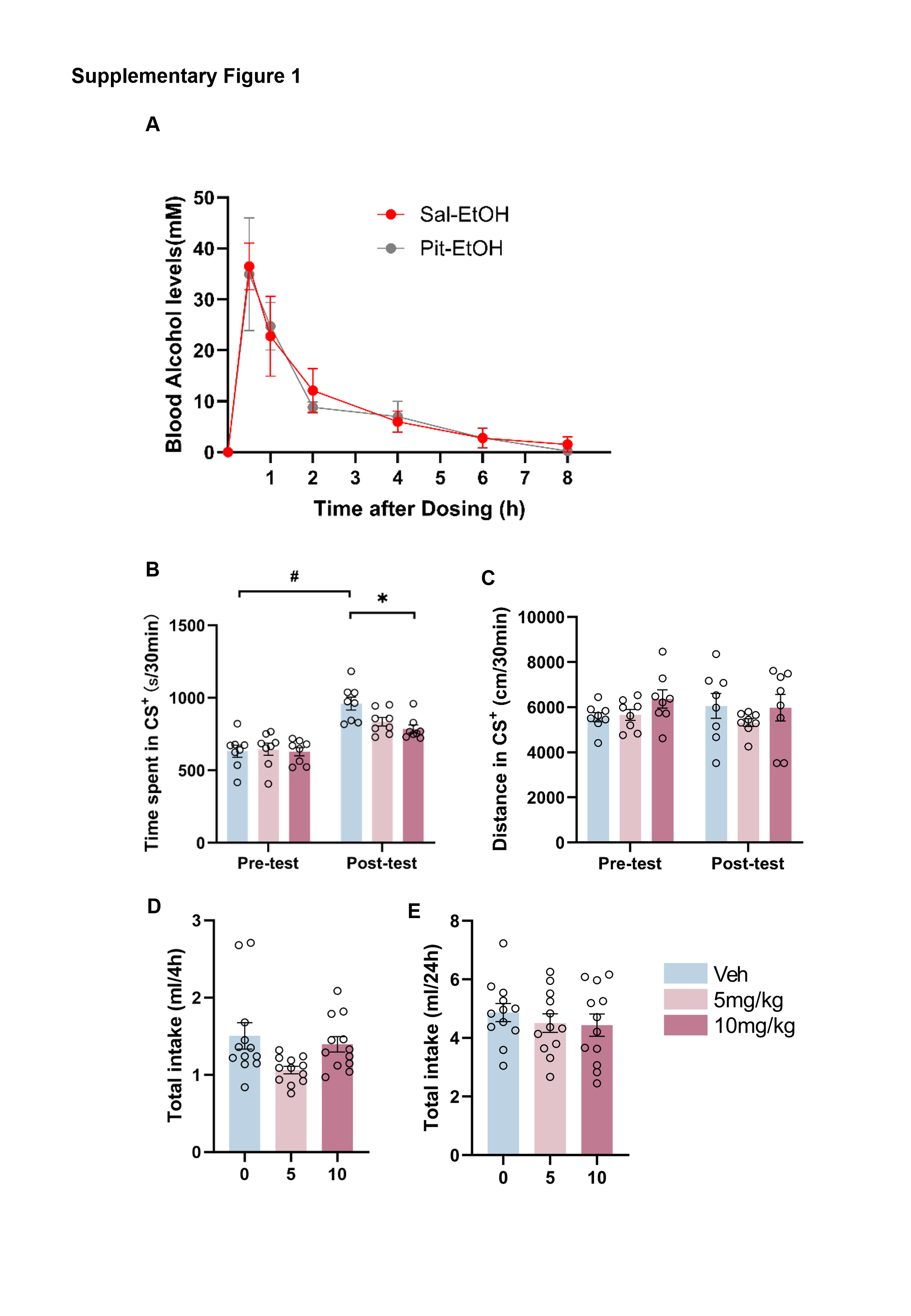


**Figure S1. Effects of pitolisant on blood alcohol concentrations, ethanol-induced conditioned place preference, locomotor activity, and alcohol consumption.** (A) Effect of pitolisant administration (10 mg/kg) on blood alcohol concentrations; (B) Time spent by mice in the ethanol-paired chamber during the pre-test and post-test day in the CPP experiment; (C) Locomotor distance of animals in the ethanol-paired chamber during the pre-test and post-test sessions after treatment; (D) Total fluid intake within the first 4 h after pitolisant injection; (E) Total fluid intake within 24 h after pitolisant injection at different doses. One-way ANOVA was used, followed by Bonferroni's multiple comparisons. **p* < 0.05, ^#^*p* < 0.05. All data are shown as mean ± SEM.


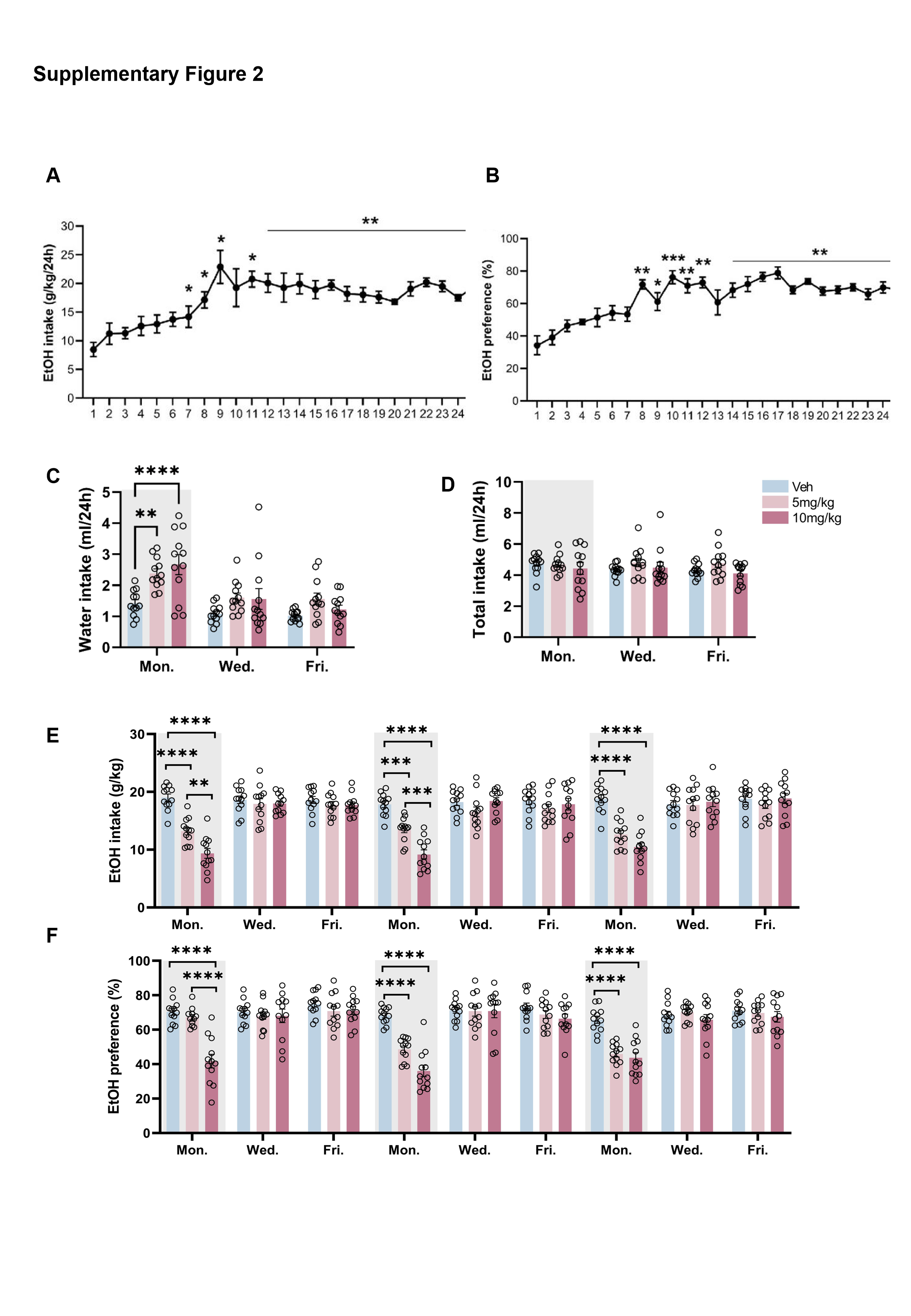


**Figure S2. Pitolisant reduces voluntary ethanol intake and preference of mice in the IA2BC model.** (A) Trend of 24 h ethanol intake during the 8-week observation period; (B) Trend of ethanol preference during the 8-week observation period; (C) Effect of systemic pitolisant administration (5 and 10 mg/kg) on 24 h water intake; (D) Effect of systemic pitolisant administration (5 and 10 mg/kg) on 24 h total fluid intake; (E) Effect of pitolisant administration (5 and 10 mg/kg) on 24 h ethanol intake; (F) Effect of pitolisant administration (5 and 10 mg/kg) on ethanol preference. Statistical analysis was conducted using one-way ANOVA followed by Bonferroni's multiple comparisons test. **p* < 0.05, ***p* < 0.01, ****p* < 0.001, *****p* < 0.0001 vs. Veh. All data are shown as mean ± SEM.


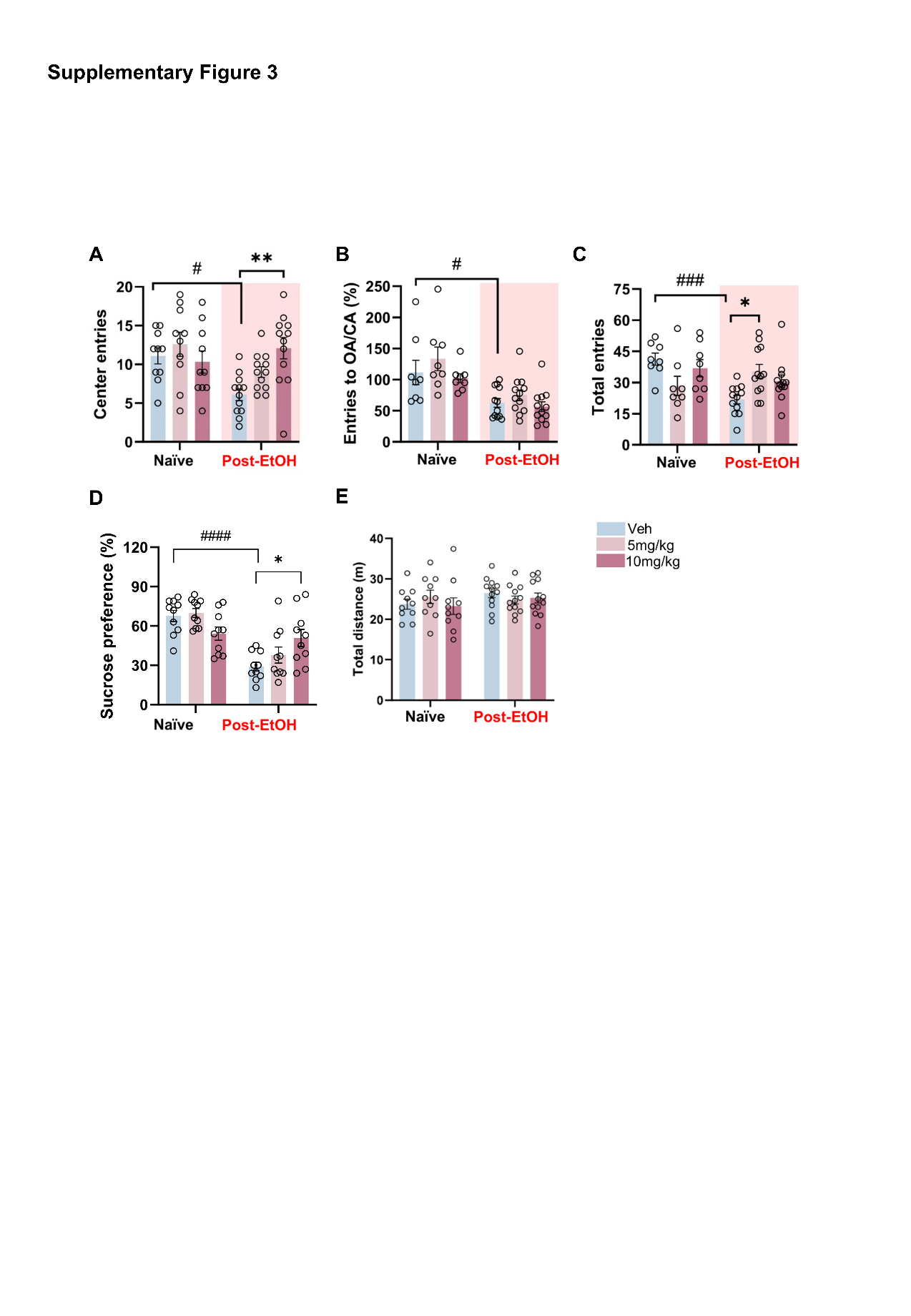


**Figure S3 The effects of different doses of pitolisant administration on fluid intake and anxiety- and depression-like behaviors in mice.** (A) Number of entries into the center area after administration of 5 and 10 mg/kg pitolisant; (B) Ratio of entries into open arms to closed arms in the elevated plus maze (EPM); (C) Total entries into different regions of the EPM; (D) Sucrose preference test (SPT). (E) Total distance after administration of 5 and 10 mg/kg pitolisant in OFT; Statistical analysis was conducted using one-way ANOVA followed by Bonferroni's multiple comparisons test. ***p* < 0.01, ****p* < 0.001, *****p* < 0.0001; ^#^*p* < 0.05, ^##^*p* < 0.01, ^###^*p* < 0.001, ^####^*p* < 0.0001. All data are shown as mean ± SEM.


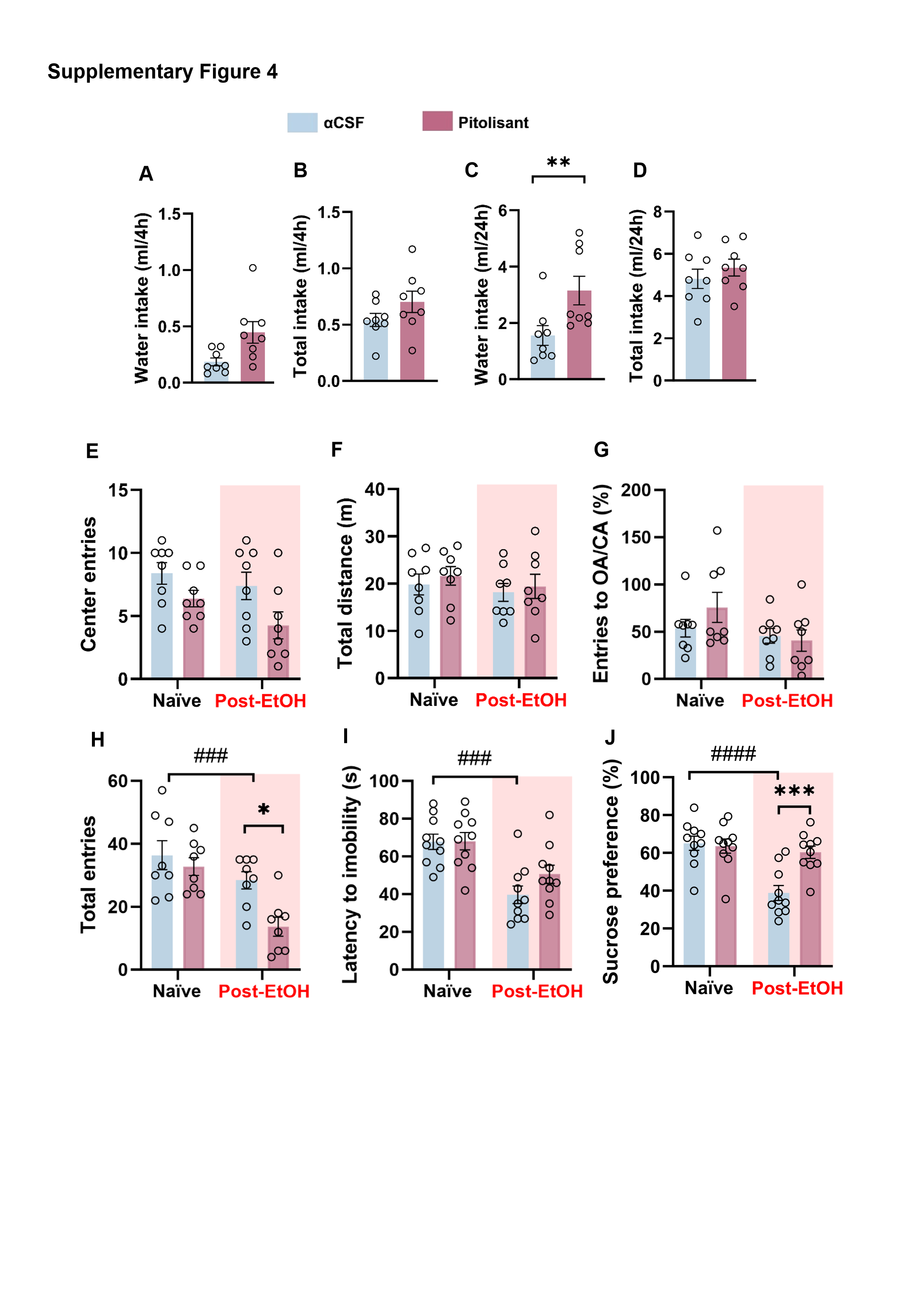


**Figure S4 Intracerebroventricular administration of pitolisant increased water intake without altering total fluid consumption and modulated anxiety- and depression-like behaviors in mice.** (A) Water intake within the first 4 h after pitolisant injection; (B) Total fluid intake in the first 4 h after pitolisant injection; (C) 24h Water intake after pitolisant injection; (D) Total fluid intake from both bottles within the 24h after pitolisant injection. Statistical analysis was performed using unpaired t-tests. (E) Number of entries into the center area by mice; (F) Locomotor distance of mice in the open field; (G) Ratio of entries in the open arms to closed arms of the EPM; (H) Total entries into the open arms and closed arms of the EPM; (I) Immobility latency of mice from the start of the TST test. (J) Sucrose preference test (SPT). Statistical analysis was performed using unpaired t-tests. ####p < 0.01. All data are shown as mean ± SEM. ***p* < 0.01. All data are shown as mean ± SEM.


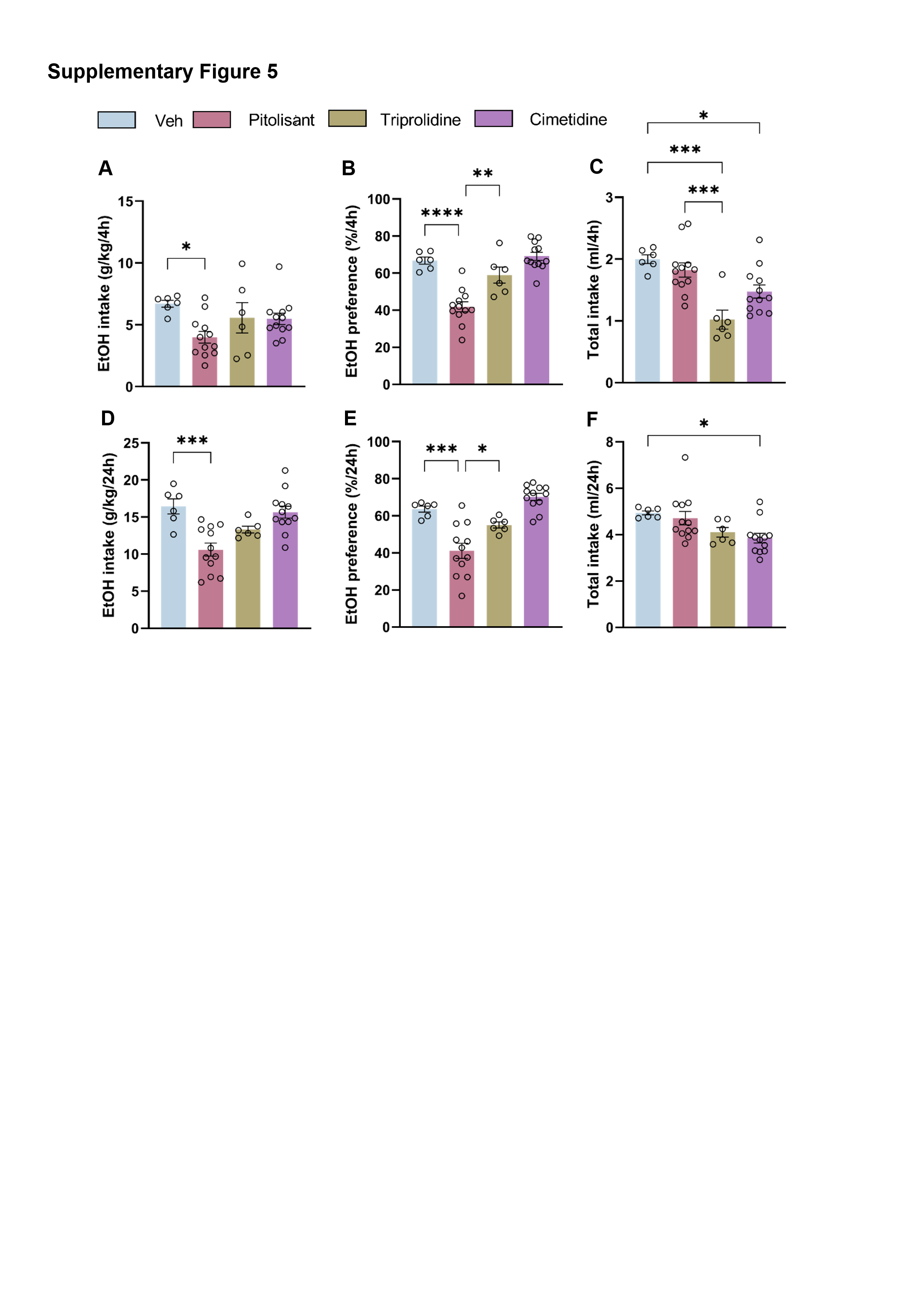


**Figure S5 Effects of intra-LHb histamine receptor antagonists alone on ethanol drinking behavior.** (A) Ethanol intake during the first 4 h after administration; (B) Ethanol preference during the first 4 h after administration; (C) Total fluid intake at 4 h post-administration; (D) 24 h ethanol intake after treatment; (E) 24 h ethanol preference after treatment; (F) Total fluid intake at 24 h post-administration. Statistical analysis was conducted using one-way ANOVA followed by Bonferroni's multiple comparisons test. **p* < 0.05, ***p* < 0.01, ****p* < 0.001, *****p* < 0.0001 vs. Veh. All data are shown as mean ± SEM.


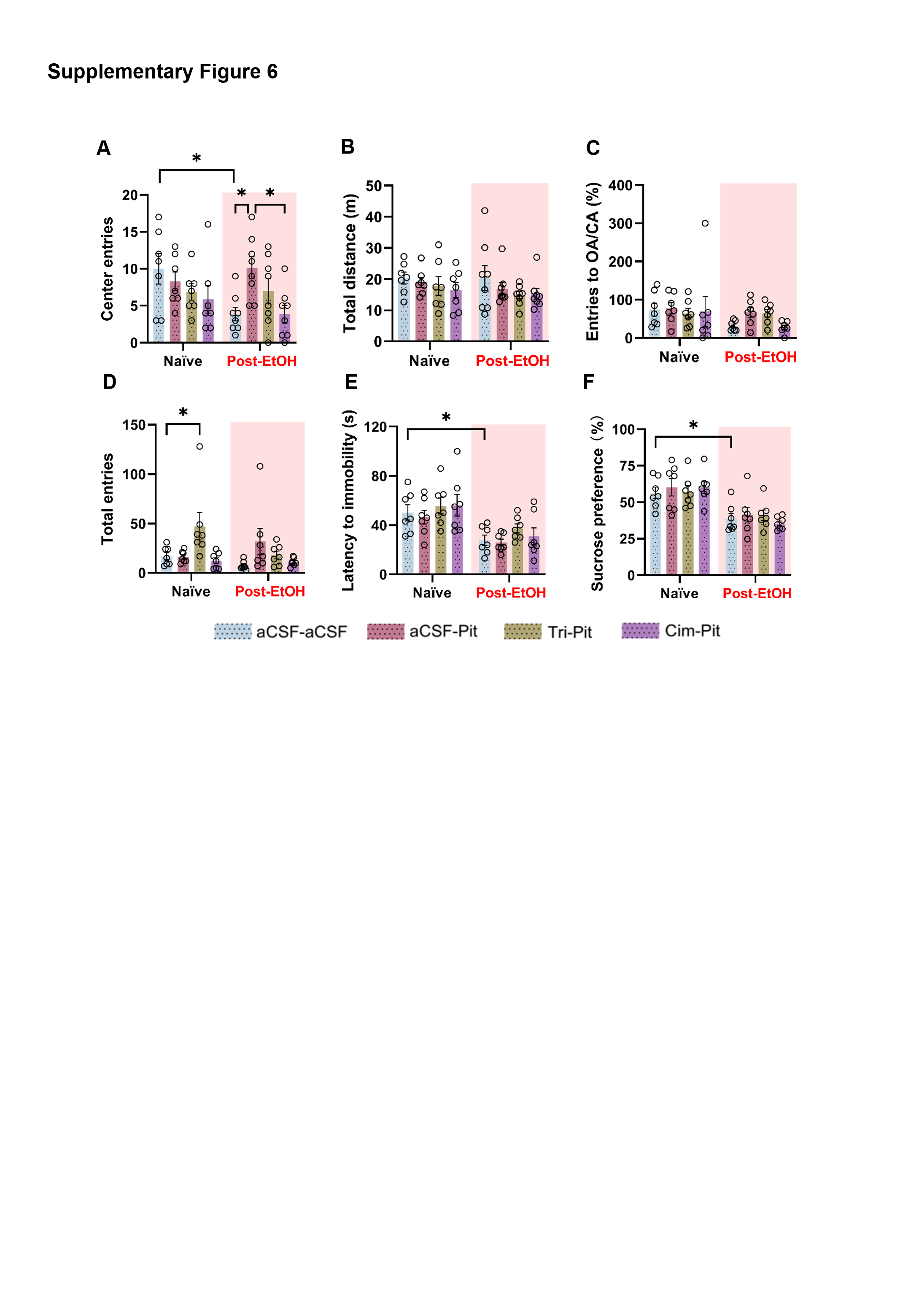


**Figure S6 Effects of intra-LHb Pitolsiant combined with H1R or H2R antagonists on anxiety- and depression-like behavior in mice.** (A) Number of entries into the center area by mice; (B) Locomotor distance of mice in the open field; (C) Ratio of time spent by mice in the open arms to closed arms of the EPM; (D) Total entries into different regions of the EPM; (E) Immobility latency of mice from the start of the TST test; (E) Sucrose preference of mice in the SPT. Statistical analysis was conducted using one-way ANOVA followed by Bonferroni's multiple comparisons test. **p* < 0.05, ***p* < 0.01, ****p* < 0.001, *****p* < 0.0001 vs. Veh. All data are shown as mean ± SEM.

**
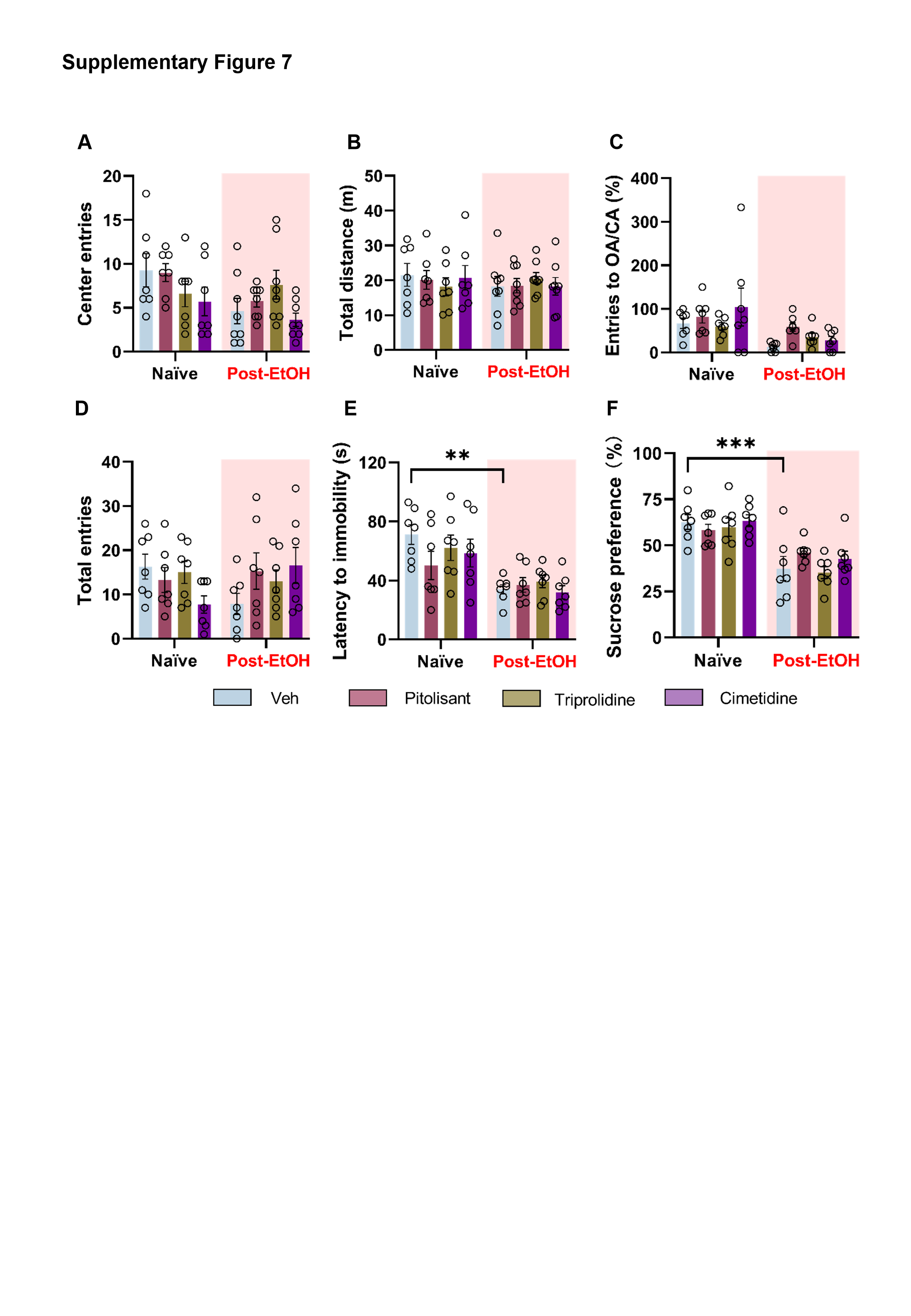
**

**Figure S7 Effects of intra-LHb histamine receptor antagonists alone on anxiety- and depression-like behavior in mice.** (A)Number of entries into the center area by mice; (B) Locomotor distance of mice in the open field; (C) Ratio of time spent by mice in the open arms to closed arms of the EPM; (D) Total entries into different regions of the EPM; (E) Immobility latency of mice from the start of the TST test; (F) Sucrose preference of mice in the SPT. Statistical analysis was conducted using one-way ANOVA followed by Bonferroni's multiple comparisons test. **p* < 0.05, ***p* < 0.01, ****p* < 0.001, *****p* < 0.0001 vs. Veh. All data are shown as mean ± SE

**Reference**

1 Sánchez-Marín L, Ladrón de Guevara-Miranda D, Mañas-Padilla MC, Alén F, Moreno-Fernández RD, Díaz-Navarro C, et al. Systemic blockade of LPA(1/3) lysophosphatidic acid receptors by ki16425 modulates the effects of ethanol on the brain and behavior. Neuropharmacology. 2018;133:189-201.

2 Ding R, Tang Y, Cao G, Mai Y, Fu Y, Ren Z, et al. Lateral habenula IL-10 controls GABA(A) receptor trafficking and modulates depression susceptibility after maternal separation. Brain, behavior, and immunity. 2024;122:122-36.

3 Cunningham CL, Howard MA, Gill SJ, Rubinstein M, Low MJ, Grandy DK. Ethanol-conditioned place preference is reduced in dopamine D2 receptor-deficient mice. Pharmacology, biochemistry, and behavior. 2000;67(4):693-9.

4 Fu Y, Li W, Mai Y, Guan J, Ding R, Hou J, et al. Association between RMTg Neuropeptide Genes and Negative Effect during Alcohol Withdrawal in Mice. International journal of molecular sciences. 2024;25(5).

5 Li W, Ren Z, Tang Y, Fu Y, Sun S, Ding R, et al. Rostromedial tegmental nucleus nociceptin/orphanin FQ (N/OFQ) signaling regulates anxiety- and depression-like behaviors in alcohol withdrawn rats. Neuropsychopharmacology : official publication of the American College of Neuropsychopharmacology. 2023;48(6):908-19.

6 Ren Z, Hou J, Li W, Tang Y, Wang M, Ding R, et al. LPA1 receptors in the lateral habenula regulate negative affective states associated with alcohol withdrawal. Neuropsychopharmacology : official publication of the American College of Neuropsychopharmacology. 2023;48(11):1567-78.

7 Cao G, Chen B, Sun Y, Qiao J, Liu T, Hou J, et al. Plasma metabolic profiles in alcohol use disorder: diagnostic role of arginine and emotional implications of N6-acetyl-lysine and succinic acid. BMC psychiatry. 2025;25(1):563.
